# Molecular signatures of divergence and selection in closely related pine taxa

**DOI:** 10.1007/s11295-018-1296-3

**Published:** 2018-10-28

**Authors:** Witold Wachowiak, Julia Zaborowska, Bartosz Łabiszak, Annika Perry, Giovanni M. Zucca, Santiago C. González-Martínez, Stephen Cavers

**Affiliations:** 1 Centre for Ecology and Hydrology Edinburgh, Bush Estate, Penicuik, Midlothian, EH26 0QB UK; 2 Institute of Dendrology, Polish Academy of Sciences, Parkowa 5, 62-035 Kórnik, Poland; 3 Institute of Environmental Biology, Faculty of Biology, Adam Mickiewicz University, Umultowska 89, 61-614 Poznań, Poland; 4 BIOGECO, INRA, Univ. Bordeaux, 33610 Cestas, France

**Keywords:** Nucleotide polymorphisms, Nuclear loci, *mt*DNA, Local adaptation, DNA sequencing, Speciation

## Abstract

Efforts to detect loci under selection in plants have mostly focussed on single species. However, assuming that intraspecific divergence may lead to speciation, comparisons of genetic variation within and among recently diverged taxa can help to locate such genes. In this study, coalescent and outlier detection methods were used to assess nucleotide polymorphism and divergence at 79 nuclear gene fragments (1212 SNPs) in 16 populations (153 individuals) of the closely related, but phenotypically and ecologically distinct, pine taxa *Pinus mugo*, *P. uliginosa* and *P. uncinata* across their European distributions. Simultaneously, mitochondrial DNA markers, which are maternally inherited in pines and distributed by seeds at short geographic distance, were used to assess genetic relationships of the focal populations and taxa. The majority of nuclear loci showed homogenous patterns of variation between the taxa due to a high number of shared SNPs and haplotypes, similar levels of polymorphism, and low net divergence. However, against this common genetic background and an overall low population structure within taxa at mitochondrial markers, we identified several genes showing signatures of selection, accompanied by significant intra- and interspecific divergence. Our results indicate that loci involved in species divergence may be involved in intraspecific local adaptation.

**Electronic supplementary material:**

The online version of this article (10.1007/s11295-018-1296-3) contains supplementary material, which is available to authorized users.

## Introduction

Identifying the molecular basis of evolution is a major challenge in biology. So far, efforts to detect loci under selection in plants have mostly focussed on single species. However, assuming that selection acting on variation within a species may eventually lead to speciation, a between-species comparative approach can improve the power to detect genes involved in adaptation (Nosil and Feder 2013). Interspecific comparisons allow for the detection of divergence at candidate loci and its assessment relative to the average divergence among species and to that at neutrally evolving genes. Comparative studies of intra- and interspecific genetic variation can also show whether the same loci contribute to adaptive variation in different species.

To date, comparative genomic studies on model plant species have reached different conclusions regarding the rate and magnitude of genomic change that drives adaptation and speciation. For instance, contrasting patterns of genomic divergence, including the size and location of regions under selection, were observed between dune and non-dune ecotypes of sunflower *Helianthus petiolaris* relative to *H. annuus* (Andrew and Rieseberg 2013). Scans of the patterns of polymorphism and divergence identified genomic regions enriched for genes involved in ion transport and metal detoxification in adaptively differentiated *Arabidopsis lyrata* (Turner et al. 2008). Overall, genome scans for adaptation and speciation genes have identified signatures of selection in 0.4 to 35.5% of the genomic regions studied in different plant species (Ahrens et al. 2018; Strasburg et al. 2012). A recent comparative genomic study suggests convergent evolution in coniferous tree species (Yeaman et al. 2016). Other studies have identified variation at a shared set of genes associated with the same environmental factor across three oak species (Rellstab et al. 2016), but mainly genomic studies of trees have focused on single species. These provide some evidence of the influence of selection on patterns of nucleotide polymorphism along environmental clines, e.g. in dehydrin genes, phytochromes and genes related to wood formation in pines, spruce, oak and poplar, suggesting polygenic control of many adaptive traits (González-Martinez et al. 2007; Ingvarsson et al. 2006; Wachowiak et al. 2009). However, research on the genes underlying adaptation and their genomic context is still at an early stage in non-model taxa, and particularly in plants with complex genomes such as the conifers. Available draft sequences of a few conifer species indicate that large genome size (commonly over 20 Gbp) results, among others, from the presence of retrotransposons and other repetitive content (Neale et al. 2014; Nystedt et al. 2013). As the whole-genome re-sequencing is currently not an option with such large genomes, the candidate-gene approach is still a reasonable approach in population genetic studies in pines.

The *Pinus mugo* complex is a group of closely related European conifer taxa. It includes *P. mugo* (Dwarf mountain pine), *P. uncinata* (mountain pine) and *P. uliginosa* (peat-bog pine). These taxa are especially suitable for comparative analyses of the pattern of polymorphism and divergence as they form a monophyletic group within Pinaceae (Grotkopp et al. 2004) that diverged within the last 5 million years (Wachowiak et al. 2011) but differ strongly in phenotype, geographical distribution and ecology, in particular for traits related to dehydrative stress and temperature (Wachowiak et al. 2013). Currently, they have largely disjunct distributions following postglacial range shifts (Critchfield and Little 1966). *Pinus mugo* is a high-altitude, polycormic species of a few meters in height, which grows above the tree line in the mountainous regions of Central and Southeastern Europe including major populations from the Alps in the west, through the Sudetes, Tatras and Carpathians to the Rila and Pirin mountains of the Balkans in the east (Critchfield and Little 1966). *Pinus uncinata* and *P. uliginosa* are trees of up to 20 m height, the former adapted to mountainous regions of western Europe including the Pyrenees, the Massif Central, Western Alps and several marginal populations in the Iberian Peninsula, the latter to peat bogs in lowland areas of Central Europe.

Despite morphological, geographical and ecological differentiation, the three taxa show high genetic similarity at biochemical (monoterpenes, isozyme) and molecular markers (Lewandowski et al. 2000) and have the same number of chromosomes (2n = 24) (Grotkopp et al. 2004). They have recently diverged and can hybridize in contact zones (Jasińska et al. 2010; Wachowiak and Prus-Głowacki 2008). Altogether, their high ecological and phenotypic diversity and similar genetic background make these taxa a suitable experimental system to search for parallel signatures of divergence and local adaptation at the genomic level.

In this study, we looked at the patterns of genetic variation within and among the focal taxa in populations’ representative of their distribution range in Europe to search for patterns of divergence and local adaptation in a set of orthologous genomic regions. Using a set of mitochondrial DNA markers, nucleotide sequence data from 79 gene fragments and a sample of 153 trees (including 79 *P. mugo*, 50 *P. uncinata* and 24 *P. uliginosa*) from 16 populations, we evaluated population structure, levels of polymorphism and divergence, and tested for the signature of selection at inter- and intraspecific levels. We asked if the genes involved in genetic divergence among taxa (i.e. speciation) were also implicated in local adaptation within taxa. We also assessed whether the same genes were under selection in different taxa, which would suggest a common role in adaptive processes.

## Material and methods

### Sampling and DNA extraction

**Fig. 1:**
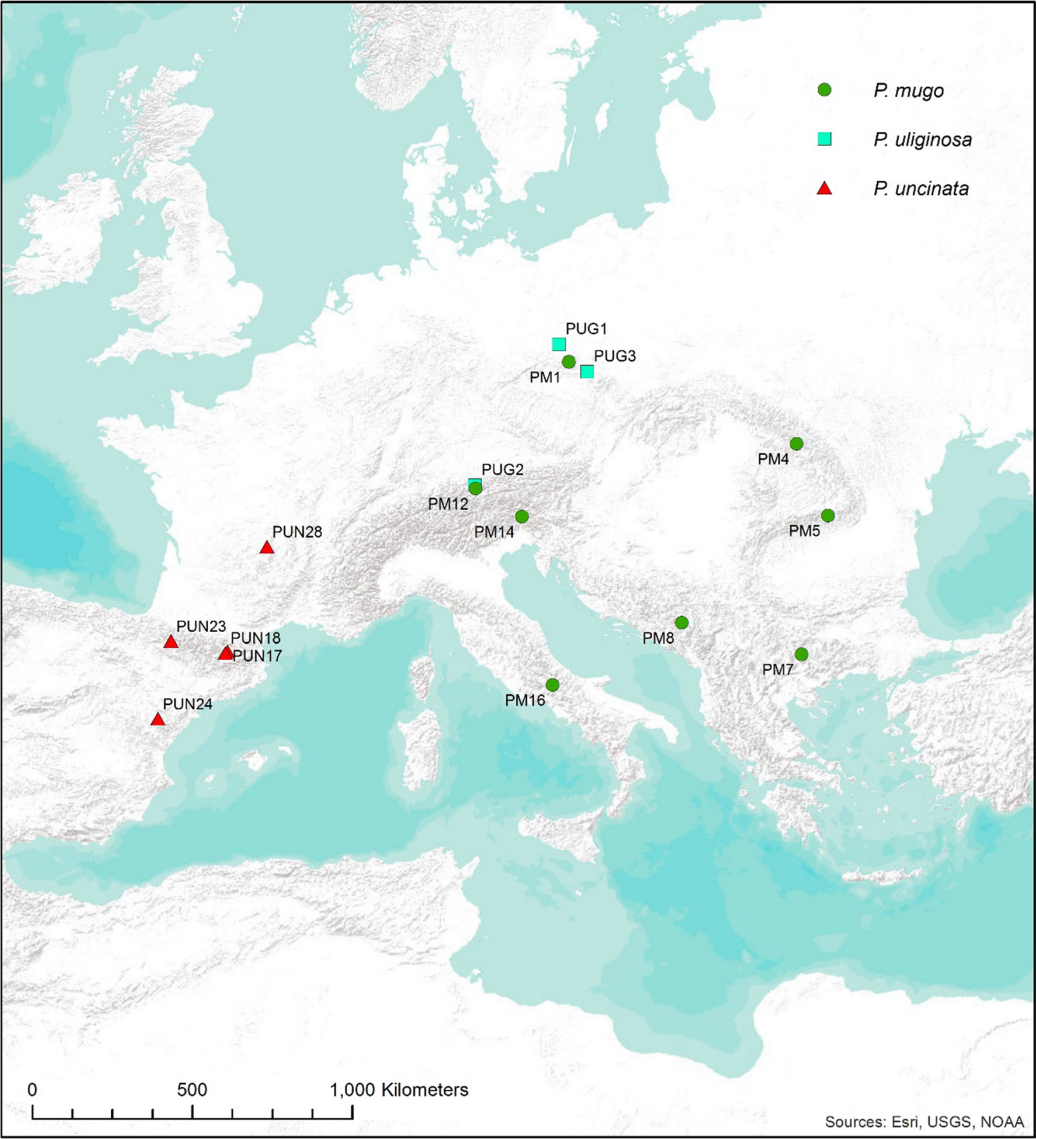
Location of the sample sites. Populations of all taxa are shown; taxa are indicated by label: PM—*Pinus mugo*, PUG—*P. uliginosa* and PUN—*P. uncinata*. Figure produced in ArcMap v10.1

Seeds were collected in each of 16 natural populations in Europe (Fig. 1); 8 for *P. mugo*, 5 for *P. uncinata* and 3 for *P. uliginosa*. We focused on allopatric stands avoiding sampling of any contact zones of the taxa. To allow comparison of similar sized samples that reflect geographical locations, we defined regional groups of populations for *P. mugo* that were labelled Central Europe (Sudetes and Alps), Carpathians (eastern and southern) and Balkans (Pirin and Durmitor Mts). A single *P. mugo* population from Italy was excluded from the group comparisons. No regional groups were constructed for *P. uliginosa* or *P. uncinata*. In total, 153 samples were analysed, comprising ten individuals from most locations except for PM14 and PUG2 (*N* = 9) and PUG3 (*N* = 5) (Table S1). Sampled individuals were separated at a distance of at least 30 m. Genomic DNA was extracted from haploid megagametophytes from germinated seeds using a DNeasy Plant Mini Kit (Qiagen, Hilden, Germany) and the yield and quality of DNA extract was evaluated using Qubit 3.0 fluorimeter (Invitrogen, UK).

### Loci, PCR and sequencing

A total of 79 gene fragments were targeted for sequencing across the taxa, which were associated with a broad range of different functions, including transcription factors, metabolic and signalling pathways (such as cold, drought and pathogen stress responses), photoperiodic response and cellular transport (Table S2). PCR primers for each nuclear gene fragment were designed based on unique cDNA originally sequenced in *P. taeda* (Mosca et al. 2012). Additionally, three mitochondrial DNA (*mt*DNA) fragments including P11 (Donnelly et al. 2017) were analysed for each individual (Table S2). Mitochondrial DNA is maternally inherited in pines, undergoes seed dispersal and is effective for characterising population structure. PCR amplification was carried out with Thermo MBS thermal cyclers (for nuclear loci) and Applied Biosystems ABI 2720 (for mitochondrial regions) in a total volume of 15 μl containing 15 ng of haploid template DNA, 10 μM of each dNTP, 0.2 μM each of forward and reverse primers, 0.15 U *Taq* DNA polymerase, 1xBSA, 1.5 μM of MgCl_2_ and 1xPCR buffer (BioLabs or Novazyme). Standard amplification procedures were used with initial denaturation at 94 °C for 3 min, followed by 35 cycles with 30 s denaturation at 94 °C, 30 s annealing at 60 °C for nuclear loci and 57 °C for *mt*DNA regions for 1 min, 30 s extension at 72 °C and a final 5-min extension at 72 °C. PCR fragments were purified using Exonuclease I-Shrimp Alkaline Phosphatase enzymatic treatment. About 20 ng of PCR product was used as a template in 10 μl Sanger sequencing reactions with the Big Dye Terminator DNA Sequencing Kit v3.1 (Applied Biosystems). Multilocus haplotypes were determined by direct sequencing of haploid DNA using 3730xl DNA Analyzer (Applied Biosystems). CodonCode Aligner (Codon Code Corporation) was used to edit and align sequences. Sequence data at nuclear genes are deposited in the NCBI repository (Table S2).

### Nucleotide variation

We looked at the overall pattern of genetic variation at intra- and interspecific levels. Nucleotide diversity of nuclear genes was measured as the average number of differences per site (π) between two sequences (Nei 1987). Multilocus estimates of the population mutation parameter, theta (θ_W_, Watterson’s 1975) were computed based on the number of total and/or silent segregating sites and the length of each locus using Markov chain Monte Carlo (MCMC) simulation, with the length of burn-in of 5000 and the number of simulated values of 100,000, under a Bayesian model (Pyhäjärvi et al. 2007). The ratio of recombination to mutation rates (*ρ*/θ) was calculated to estimate their relative influence on patterns of diversity in each taxon and in regional groups. The non-linear least squares estimate of *ρ* (*ρ* = 4N_e_c, were *N*_e_ is the effective population size and *c* is the recombination rate) was fitted by the *nls*-function implemented in *R* (www.r-project.org) based on the correlation coefficient *r*^2^ between polymorphic informative sites and the distance in base pairs (bp) between sites. Locus-by-locus estimates of net divergence between taxa (Nei 1987), and the number of shared, exclusive and fixed polymorphic sites and haplotypes for each locus were determined using SITES 1.1 (Hey and Wakeley 1997). Sequences from *P. taeda* were obtained from GenBank for outgroup comparisons. The number of haplotypes (*N*) and haplotype diversity (*H*_d_) were computed for each gene using DnaSP v5 (Librado and Rozas 2009). The number and frequency of unique and shared haplotypes (the entire length of the sequenced gene fragments) in pairwise comparisons between taxa was calculated with Arlequin v.3.5 (Excoffier and Lischer 2010).

### Population structure and differentiation

Genetic distance based on all polymorphic *mt*DNA sites was calculated and used in principal coordinate analysis (PCoA) to show genetic relationships between populations and taxa. Genetic relationships between samples at nuclear loci were explored using BAPS 6.0 (Corander and Tang 2007) and STRUCTURE 2.2 (Pritchard et al. 2000), for values of *K* from 1 to 15 with estimates averaged over ten independent runs in each case, with optimal *K* determined from likelihood and assignment proportions. For BAPS 6.0, datasets comprising all samples were tested. The Codon linkage model was selected, with 100 iterations to estimate admixture coefficients, 100 reference individuals and 10 iterations to estimate admixture coefficients for the reference individuals. For STRUCTURE 2.2, datasets including all polymorphic sites, and excluding linked sites as determined by a significant Fisher’s exact test after Bonferroni correction, were tested. The correlated allele frequencies model was used; burn-in was set to 100,000 and run length to 1,000,000. To further assess among-population differentiation, we used principal coordinate analysis (PCoA) based on the mean net distance between populations across all SNPs, estimated with the Jukes-Cantor nucleotide substitution model with pairwise deletion. The hierarchical distribution of multilocus genetic variation among taxa, regional groups and populations was estimated using an analysis of molecular variance (AMOVA) with Arlequin v.3.5 (Excoffier and Lischer 2010).

### Neutrality tests

Multilocus Tajima’s *D* (Tajima 1989) was computed using the difference between two distinct estimates of the scaled mutation parameter theta (θ) for each locus. Statistical significance of the test was evaluated by comparison to a distribution generated by 1000 coalescent simulations using HKA software (Jiggins et al. 2008). Deviations from neutrality at individual loci were estimated using two compound neutrality tests that were robust to demographic processes, HEW and DHEW (Zeng et al. 2007). Significance levels were determined by 10,000 coalescent simulations based on Watterson’s estimator of θ as implemented in the DH software (Zeng et al. 2007). The distribution of the test statistic was investigated for each locus in all samples from each taxon. The relationship between polymorphism and divergence was evaluated using the HKA test (Hudson et al. 1987).

### Signatures of selection and divergence

First, genetic differentiation in pairwise comparisons within and between taxa was studied locus-by-locus at both haplotype and SNP/indel level. Significance was estimated by 1000 permutations of the samples between populations, regional groups and taxa using Arlequin v.3.5. The false discovery rate (FDR) adjustment for multiple testing was conducted using the *q* value package in *R* (Bass et al. 2015) (lambda = 0.15, FDR level = 0.01). The estimates of the overall proportion of true null hypotheses (pi0) and the *q* values (that reflect the expected proportion of false positives among significant results) were calculated based on the distribution of *p* values for the set of tests conducted (Storey and Tibshirani 2003). Among-group and among-population diversity was estimated by the nearest neighbour statistic, *S*_nn_ (Lynch and Crease 1990), and tested for significance using 1000 permutations, where samples were randomly assigned to groups. Second, the full SNP dataset was used to test for outlier loci among populations using Bayesian hierarchical analysis (Foll and Gaggiotti 2008). In the first approach, coalescent simulations were used to estimate a null distribution and confidence intervals around the observed values, which allowed for the identification of outliers by locus-specific *F*_ST_ conditioned on the multilocus distribution of *F*_ST_ values. Outliers that were highly differentiated relative to background variation may indicate a locus under directional selection. Each simulated group consisted of 100 subpopulations, and 20,000 replicates of the coalescent were used to identify the expected distribution of *F*_ST_. The significance thresholds of the *F*_ST_ values were set at 95% and 99% from the simulated data using Arlequin v.3.5. For the second approach, we used BayeScan (Foll and Gaggiotti 2008) for detection of outliers, under a natural selection vs. no selection model based on differences in allele frequencies between populations. We tested for outliers with *q* value threshold of 5% using SNPs whose minor allele frequency (MAF) was greater than 0.05. A sample size of 5000 was used, with thinning intervals of 10, and 20 pilot runs each of 5000, default prior odds of 10 and burn-in of 50,000. Finally, genes that showed evidence of selection within taxa (neutrality tests, outlier detection across populations) were compared with those that showed significant divergence among taxa (outlier detection among taxa) to identify parallel signatures of adaptation at different scales (local adaptation vs. speciation). Genes that showed significant deviation from neutrality, significant between taxon divergence and a signature of selection within taxa (considering both haplotypes and SNPs) were identified.

## Results

### Nucleotide and haplotype variation

From the 79 loci, about 33 kbp were aligned across taxa, providing a set of 1212 polymorphic sites. The taxa showed high genetic similarity at the level of nucleotide polymorphism, recombination rate and net divergence. The average π_tot_ = 0.0039–0.0047 and multilocus silent theta θ_sil_ = 0.0049–0.0064 indicated no significant difference in the amount of nucleotide diversity between taxa or regional groups (Table 1).

**Table 1:** Nucleotide and haplotype diversity at 79 gene fragments in the pine taxa and regional groups defined for *Pinus mugo*

Taxa				Nucleotide diversity							Haplotype diversity		
	*N*	*L*_total_	*S*	*S*_g_	π _total_	*L*_silent_	*S*_sil_	π_silent_	θ (CI)	*ρ* (SE)	*D*	*N*	*H*_d_ (SD)
*P. mugo*	79	33,186	872	319	0.0040	21370	679	0.0053	0.0064 (0.0058–0.0070)	0.0345 (0.0026)	− 0.831^*^	9.5	0.589 (0.045)
*P. uliginosa*	24	33,164	575	185	0.0046	21,402	455	0.0061	0.0059 (0.0052–0.0066)	0.0028 (0.0005)	− 0.357^*^	5.5	0.648 (0.077)
*P. uncinata*	50	33,121	718	203	0.0047	21,281	559	0.0062	0.0059 (0.0053–0.0066)	0.0184 (0.0015)	− 0.243^*^	7.2	0.636 (0.052)
Regional groups													
PMCE	29	32,756	574	201	0.0042	21,267	457	0.0055	0.0056 (0.0049–0.0063)	0.0039 (0.0006)	− 0.488^*^	5.7	0.595 (0.073)
PMCP	20	32,784	460	171	0.0039	21,271	351	0.0049	0.0049 (0.0042–0.0056)	0.0011 (0.0005)	− 0.479^*^	4.7	0.540 (0.087)
PMB	20	32,748	477	182	0.0040	21,280	380	0.0053	0.0052 (0.0045–0.0059)	0.0046 (0.0008)	− 0.445^*^	4.7	0.585 (0.082)

Regional groups: *PMCE*, *Pinus mugo* Central Europe; *PMCP*, *P. mugo* Carpathians; *PMB*, *P. mugo* Balkans. *N*, sample size; *L*, length of sequence in base pairs; *S*, number of segregating sites; *S*_g_, number of singletons; *π*, nucleotide diversity (Nei 1987); *θ*, median Watterson’s scaled mutation for silent sites (95% credibility intervals); *ρ*, recombination rate parameter (standard error); *D*, Tajima’s *D* test (Tajima 1989); *N*, number of haplotypes; *H*_d_, haplotype diversity (standard deviation); ^*^P < 0.05

The level of polymorphism at individual loci was similar across taxa, although levels among loci differed from 0.0006 to 0.0189 (Fig. S1). The ratio of recombination to diversity was reduced in *P. uliginosa* (*ρ*/θ = 0.5) relative to *P. mugo* (*ρ*/θ = 5.4) and *P. uncinata* (*ρ*/θ = 3.1), respectively. Variation in net divergence found at individual loci ranged from 0 to 0.0062 (Fig. S2). However, low overall average net divergence (< 0.001) was found among taxa and regional groups (Table S3). Net divergence from the outgroup *P. taeda* was constant for all taxa (~ 0.024) (Table S3)*.* There were no fixed differences between the taxa at any locus and they shared 59% (*P. mugo* vs. *P. uncinata*), 63% (*P. mugo* vs. *P. uliginosa*) and 69% (*Pinus uliginosa* vs. *P uncinata*) of the polymorphic sites (Table S4). Unique haplotypes were found in all taxa (ranging from 29 to 60%) and regional groups (35–52%) (Table S5). The average haplotype diversity was very similar for each taxon (*H*_d_ = 0.59–0.65) and for regional groups of populations (*H*_d_ = 0.54–0.60) (Table 1).

### Population structure and differentiation

**Fig. 2:**
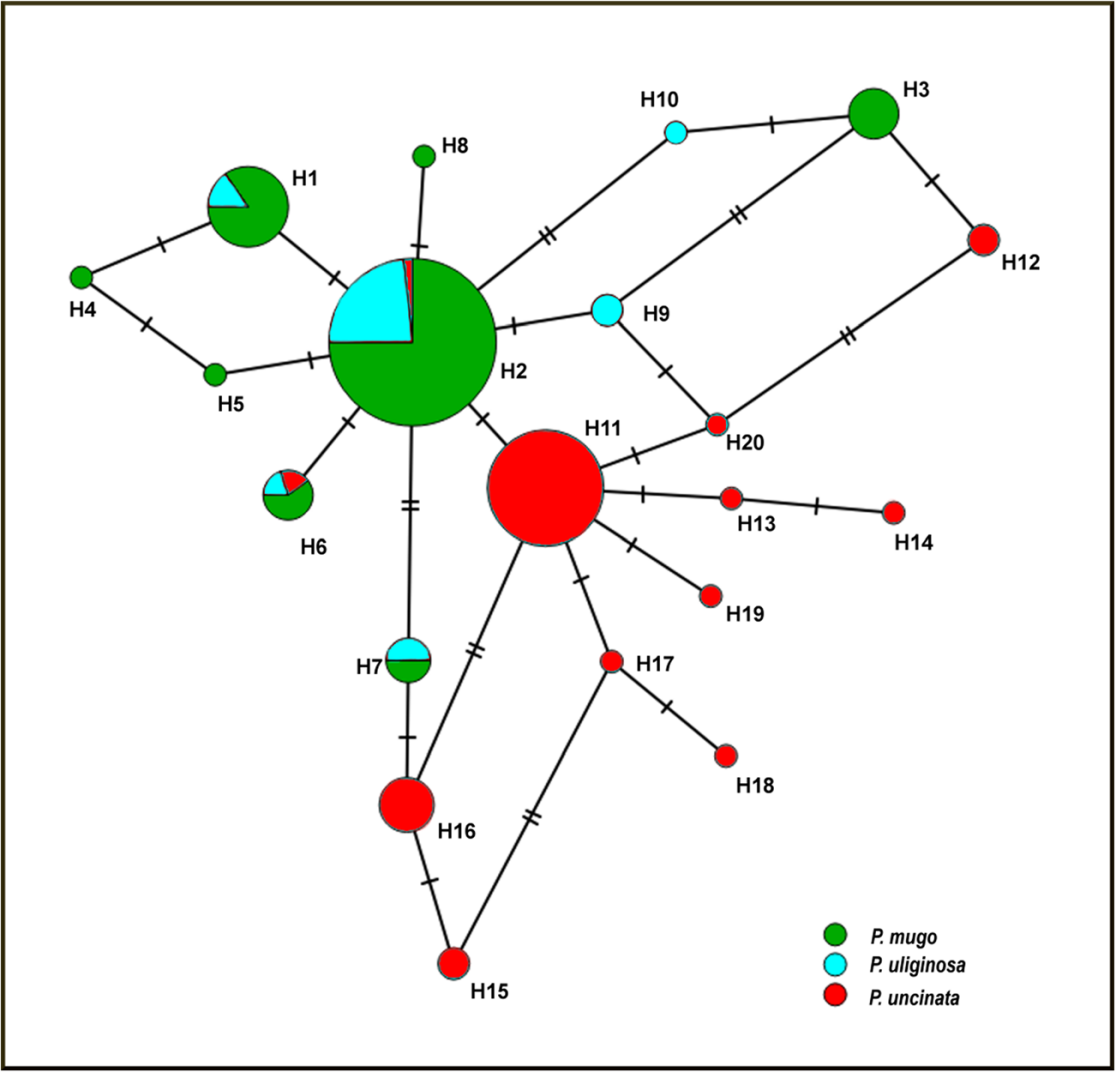
Network of haplotypes detected at three *mt*DNA regions in the taxa from the *Pinus mugo* complex. Areas of the circles are proportional to haplotype frequencies, hatch marks represent numbers of nucleotide differences between them and shading indicates taxa

**Fig. 3:**
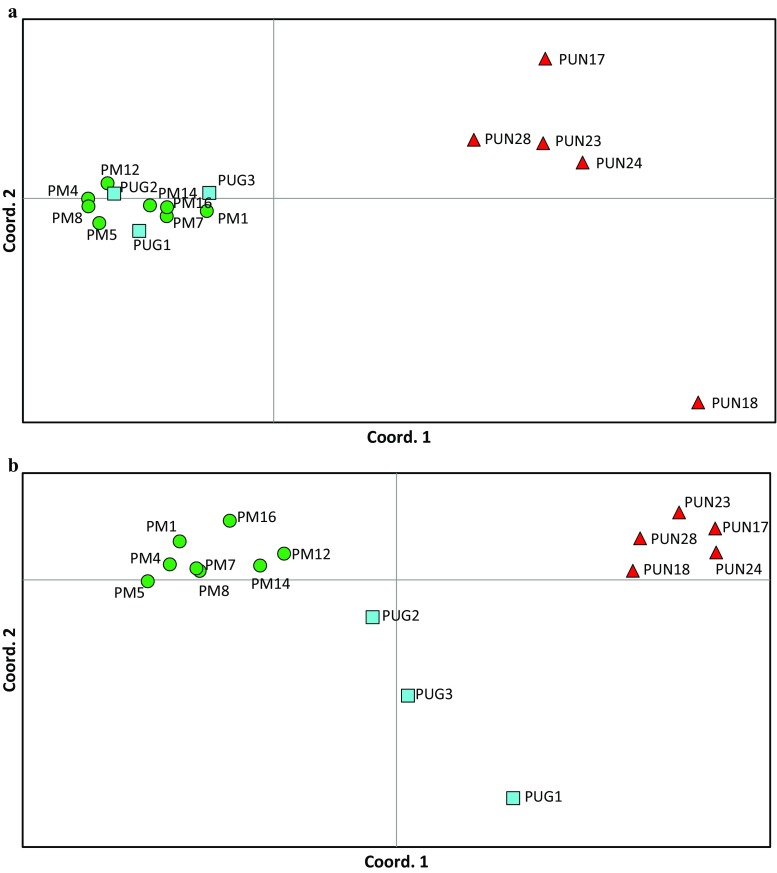
Principal coordinate analysis based on genetic distance among populations at polymorphic sites in **a** the mitochondrial genome and **b** nuclear genes. *Pinus mugo* ; *P. uliginosa*  and *P. uncinata*

**Fig. 4:**
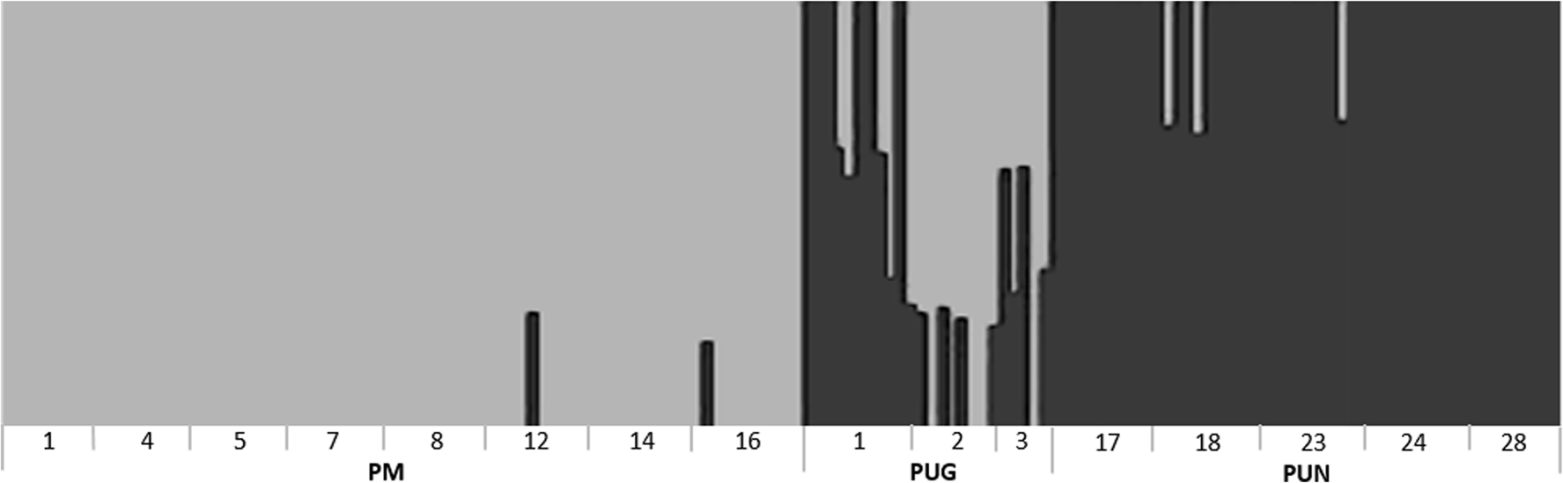
Bar plot representing the three taxa from the *Pinus mugo* complex at nuclear polymorphic sites from cluster analysis in BAPS (Corander and Tang 2007). The grey and black colours represent proportional assignment of individuals to different clusters (*K* = 2). Some evidence of admixed individuals (*P* < 0.01), indicated as two-colour bars, was found in *P. mugo*—PM12 (1 individual), PM16 (1) and *P. uncinata*: PUN18 (2), PUN23 (1). Mixed genetic constitution was found in all *P. uliginosa* populations (PUG1 (4), PUG2 (5), PUG3(4); population numbers and acronyms as in Supplementary Table S1)

The *mt*DNA loci provided a set of 13 SNPs resulting in 20 haplotypes that were predominantly partitioned between *P. mugo* and *P. uliginosa* as a group, vs. *P. uncinata* (Fig. 2; Table S6). Within *P. mugo* and *P. uliginosa*, there was a low within-taxon population structure, whilst *P. uncinata* showed more genetic variation between populations especially on the second principle coordinate axis (33% and 12% of the total variation explained by the first and the second axis, respectively; Fig. 3a). From nuclear genes, *P. uliginosa* showed intermediate variation as compared to two clearly separated groups of *P. mugo* and *P. uncinata* (27% and 16% of the total variation explained by the first and the second axis, respectively; Fig. 3b). The cluster analysis at nuclear genes suggested the presence of only two groups (*K* = 2) (Appendix 1b), with a clear division between a group containing *P. mugo* and *P. uncinata*. Only few samples in those taxa were identified as potentially admixed (Fig. 4). In contrast, *Pinus uliginosa* showed mixed genetic constitution with mosaic patterns of divergence from both clusters.

In a hierarchical AMOVA, 11% of the variation was due to differentiation between taxa (Table S7). At the intrataxon level, most variation was found within populations ranging from 93% in *P. uliginosa* to 96% in *P. uncinata* (Table S7). Significant differences between taxa were found at all polymorphic sites with *F*_ST_ values of 0.154 for *P. mugo* vs. *P. uncinata*, 0.082 for *P. mugo* vs. *P. uliginosa* and 0.075 for *P. uliginosa* vs. *P. uncinata* (*p* < 0.01). There was low but significant differentiation among regional groups in *P. mugo* (*F*_ST_ = 0.02–0.03, *p* < 0.01).

### Neutrality tests

An excess of singleton mutations across genes was indicated as significantly negative multilocus Tajima’s *D* for all taxa (*D* = − 0.831 to − 0.243; *p* < 0.05), regional groups of *P. mugo* and most individual populations except for *P. uncinata* (Table 1). Both compound neutrality tests (HEW and DHEW) provided evidence of selection at 13 loci in *P. mugo*, 7 in *P. uliginosa* and 4 in *P. uncinata* (Table 2; Table S8). Overall, there was a positive correlation between polymorphism and divergence at the genes in all analysed taxa in the HKA test (Table S9).

**Table 2:** Loci under selection based on the compound neutrality tests and the outlier patterns of intra- and interspecies polymorphisms at the three pine taxa. Numbers correspond to the loci that showed: 1, significant *F*_st_ (*p* < 0.01) at interspecies level; 2, significant *F*_st_ (*p* < 0.01) among populations within taxa; 3, significant *S*_nn_ values within taxa; 4, loci with highly diverged SNPs at interspecific level; 5, outlier SNPs within taxa; 6, significant HEW and/or DHEW tests (*p* < 0.05). I, *Pinus mugo*; II, *P. uliginosa*; III, *P. uncinata*

Acronym	Gene	*P. mugo*	*P. uliginosa*	*P. uncinata*
Pr1_11	Putative glucuronidase 3 [M]	1^III^, 6^HEW, DHEW^		
Pr1_28	Glutamate transporter [T]	4^III^, 6^HEW^	3, 4^III^, 6^HEW^	
Pr1_40	Homeobox domain containing - Protein [ST]	1^II,III^,4^III^, 6^HEW, DHEW^		
Pr1_46	Alpha-*N*-acetylglucosaminidase [M]	1^II,III^,2,3^CE-BC^ 4^II,III^,5, 6^HEW, DHEW^	1^I,III^,4^I^, 6^HEW^	
Pr2_3	2–3 ethylene-responsive transcription factor 1B-like [E]		1^III^, 6^HEW^	
Pr2_42	Transducin/WD40 domain-containing protein [ST]	1^II,III^,4^II,III^, 6^HEW^		
Pr2_44	DNA repair helicase XPB1-like [M]		1^I^,2,3,5,6^HEW^	
175	Unnamed gene [UN]	1^III^, 6^HEW, DHEW^		
ccoam	Caffeoyl CoA *O*-methyltransferase [M]	6^HEW, DHEW^		1^II^,6^HEW, DHEW^
phy	Putative phytocyanin [T]	1^II,III^,2,3^CE-BC^,4^II,II^,5, 6^DHEW^		
phytP	Phytochrome P [E]	1^III^, 6^HEW, DHEW^		
rps10	Ribosomal protein S10 [M]	1^III^, 6^HEW^		
Pr4-1	Peroxidase [ST]		5,6^DHEW^	1^I^, 6^HEW^
Pr4-5	Calcium-dependent proteokinase [ST]	1^III^,3^CE-C^,5, 6^HEW, DHEW^	6^HEW^	
Pr4-27	Putative auxin–induced transcription factor [E]	6^HEW, DHEW^	1^III^, 6^HEW, DHEW^	

Gene categories: E, expression regulation; M, metabolism; ST, signal transduction; T, transport; UN, unknown. See text and supplementary materials for details including full set of loci studied

### Signatures of divergence and selection

A similar level of genetic diversity across the taxa was accompanied by significant differentiation among taxa at several individual genes (Table 2; Table S10). The analysis of the false discovery rate for multiple testing showed very low probability that significant tests at *p* < 0.01 were false positive, with *q* values < 0.05 in most cases (Table S11). Correspondingly, the expected proportion of significant tests (1, pi0) was generally high ranging from 0.11 to 0.68 (Table S10). Overall, the highest divergence at individual loci and SNPs was observed between *P. mugo* and *P. uncinata* (Tables S10 and S12). Using BayeScan, evidence for selection was found in *P. mugo* in putative phytocyanin **(***phy*). Some of the highly diverged polymorphic sites also showed differentiation in frequency spectra within taxa, compared to the majority of SNPs (Table S13). Using the outlier detection approach, 27 SNPs were identified in 19 genes in *P. mugo*, 12 SNPs in 7 genes in *P. uliginosa* and 9 SNPs in 6 genes in *P. uncinata* (Table S13). Most of the loci that showed evidence of significant allelic frequency difference between populations or geographical groups within taxa in *P. mugo* and *P. uliginosa* (six out of seven and three out of four loci, respectively (Tables S10 and S13)) also showed evidence of significant between taxon divergence (Tables S10 and S13). Of those genes showing any deviation from neutrality in compound neutrality tests, 3 genes in *P. mugo* (including alpha-*N*-acetylglucosaminidase, phytocyanin and calcium-dependent proteokinase) and 2 in *P. uliginosa* (including glutamate transporter and DNA repair helicase) were significantly differentiated both within and among taxa. These genes are involved in signal transduction, transport and cellular metabolism and appear to be of high importance in both speciation and intraspecific local adaptation (Table 2).

## Discussion

### Polymorphism and divergence

We used 79 gene fragments to look at within- and among-taxon nucleotide variation in three European pine taxa, all of which had similar levels of nucleotide and haplotype polymorphism. The similarity between the taxa is reflected in genome organisation (in each case, 2n = 24), genome size (~ 20 × 10^3^ Mbp) and low differentiation at RAPD markers, chloroplast microsatellite loci and nuclear genes (Grotkopp et al. 2004; Heuertz et al. 2010). We found no fixed differences at any locus; the taxa shared 59–69% of SNPs, 46–56% of *mt*DNA haplotypes, showed low overall net divergence (< 0.001) and similar net divergence from *P. taeda* (0.023). This genetic similarity is likely due to recent speciation history (Wachowiak et al. 2011). As pine species are predominantly outcrossing long-lived organisms with long generation times, efficient gene flow and large effective population sizes, the time since divergence has probably been too short for genetic drift to generate significant interspecific differences resulting in a high level of shared ancestral polymorphism.

High genetic similarity may be accounted for by shared ancestry, but may also result from gene exchange during and after speciation (Yatabe et al. 2007). Successful controlled crosses, hybrid seeds and trees resulting from gene flow between species in areas of sympatric occurrence indicate that reproductive isolation between the three taxa is not complete (Wachowiak and Prus-Głowacki 2008). As shown from the geographical distribution of mitochondrial markers, there is short-distance seed-mediated gene flow, especially for *P. uncinata* and *P. mugo*, and present-day gene flow between the taxa is restricted to narrow contact zones (Jasińska et al. 2010; Wachowiak et al. 2013). In our study, only a few trees from four populations of the taxa showed some signatures of admixture. However, considering the lack of reproductive barriers and historical overlaps in range during the Pleistocene migrations related to climate oscillations, it is highly probable that hybridization has played an important role in the evolution of these taxa. Speciation through hybridization has been postulated in *P. uliginosa*, based on high genetic similarity (antigens, isozymes) and “intermediate” biometric characteristics (needle and cone traits) as compared to other closely related pine taxa (Lewandowski et al. 2000). Previous nucleotide diversity studies based on a much smaller sample size and number of loci suggested an allopatric speciation model for *P. uliginosa* with possible introgression of adaptively important loci (Wachowiak et al. 2011). The current data suggests the mixed genetic constitution with mosaic patterns of divergence from *P. mugo and P. uncinata*. Tests of alternative speciation models would be needed to verify if speciation through hybridization and/or introgression of key diverged loci could have promoted the spread of the taxon in peat-bog environments not optimal for putative parental taxa of other closely related pines. Hybrid speciation models have been postulated for other species including *P. densata* (Song et al. 2003), sunflowers (Rieseberg et al. 2003) and Ragwort (*Senecio*) (Brennan et al. 2012).

Interspecific differentiation should increase with time, given the establishment of reproductive barriers (i.e. preventing gene flow), due to accumulation of new mutations and loss of shared ancestral polymorphism (Nei 1987). Therefore, the number of highly divergent regions in the genome usually increases with increasing divergence between taxa (Martin et al. 2013). Despite similar levels of overall genetic variation, the taxa in this study were distinctive. Mitochondrial DNA could easily separate *P. mugo* and *P. uncinata*. The markers showed also high genetic similarity between *P. uliginosa* and *P. mugo*. However, based on *mt*DNA makers and previous work (Wachowiak et al. 2011, 2013), it was possible to separate *P. mugo* and *P. uliginosa* at nuclear genes, some of which clearly diverged between taxa. *Pinus uliginosa* and *P. uncinata* showed only weak genetic differentiation at nuclear loci, with the largest number of shared SNPs and haplotypes. Our estimates of the genetic relationships between taxa supported those inferred in previous isozyme and biometric reports, indicating a close relationship between *P. mugo*, *P. uliginosa* and *P. uncinata* (Lewandowski et al. 2000). Some evidence of ongoing divergence has been found in karyotype studies of heterochromatin patterns between *P. mugo* and *P. uncinata* (Bogunić et al. 2011), which showed marginal average net divergence (0.0004) in our data set.

### Population structure

Our results indicate low structure among *P. mugo* and *P. uliginosa* populations at *mt*DNA loci. Some evidence of differentiation was found in *P. uncinata* with a single outlier population from Andorra. In all taxa, overall population structure at nuclear genes was weak, with 93–98% of variation present within populations. The generally low among-population differentiation at neutral nuclear markers found in conifers is usually attributed to wind pollination and efficient gene flow over large geographical distances. Patterns of nucleotide variation also suggested population size expansion. An excess of low-frequency mutations (i.e. negative Tajima’s *D*) and very similar levels of nucleotide polymorphism were observed in each taxon and most individual populations. Given the geographical isolation of the populations, the overall genetic similarity between populations could be due to shared recolonization history, recent fragmentation of populations or efficient long range, primarily pollen-mediated gene flow (Varis et al. 2009). However, higher resolution neutral markers are needed to conclusively disentangle the genetic relationships and population structure of the taxa.

### Genes for local adaptation and speciation

Loci under selection should show allele frequency differences contrasting background variation. Directional selection on standing genetic variation at loci involved in local adaptation or speciation would result in a reduction of shared polymorphisms and increased divergence between populations/species. Over time, exclusive, high-frequency polymorphisms should accumulate compared to the random segregation of ancestral polymorphisms at selectively neutral loci (Excoffier 2004). The chances of detecting such signatures are maximised by sampling widely across the geographic range, assuming populations are differentially adapted due to selection driven by a number of factors including climate and biotic interactions.

Overall, we found more evidence for between- than within-taxon divergence. Such a result is expected under ecological speciation models for which barriers to gene flow evolve between populations and species as a result of ecologically based divergent selection (Rundle and Nosil 2005). Several loci showed evidence for selection in neutrality tests and both intra- and interspecific differentiation against the background genetic variation. In *P. mugo* and *P. uliginosa*, a total of five genes showed evidence of selection and intraspecific outlier patterns of variation. These included genes involved in cellular metabolism in both taxa, plus transport and signal transduction in *P. mugo*. However, none of the genes showing signatures of selection within taxa were common to all taxa. In other studies, evidence for molecular adaptation in both *P. pinaster* and *P. halepensis* was found at one gene (4-coumarate: CoA ligase), whilst more genes showed taxon-specific departures from neutrality (Grivet et al. 2011).

In general, more genes departing from neutrality were found in *P. mugo* than in *P. uncinata* and *P. uliginosa* (Table 2). In our study, *P. mugo* populations covered much broader geographical areas exposed to a wider range of testing conditions as compared to the two other taxa. Unique patterns of intraspecific selection and divergence were found in *P. mugo* at ten loci including genes related to regulation of gene expression, cellular transport and metabolism. A subset of these genes also showed signatures of directional selection as reduced diversity within populations and higher linkage disequilibrium. Such a pattern of variation is expected for loci under selection that favours certain alleles underlying fitness-related adaptation. In previous genomic studies focusing on plant speciation, the proportion of loci identified as outliers ranged from 0.4 to 35.5% (Strasburg et al. 2012). For instance, 3.4% of outliers for divergence were found in *Helianthus annuus* and *H. debilis* (Ahrens et al. 2018; Scascitelli et al. 2010), 5–28% in *Quercus robur* and *Q. petraea* (Guichoux et al. 2013) and about 30% in hybrid zones of *Populus alba* and *P. tremula* (Lexer et al. 2010).

In our dataset, the majority of loci that showed both evidence of selection in compound neutrality tests and an outlier pattern of differentiation at the within-taxon level also showed evidence of significant between-taxon divergence. Therefore, in part, selection has operated on both evolutionary and more recent, ecological timescales. However, not all genes that showed significant patterns of between-taxa divergence also showed evidence of selection within taxa and vice versa. Our study provides no evidence on convergent local adaptation among these closely related taxa. We found no gene that showed evidence of selection both within and between taxa, which may suggest that different loci play a role in local adaptation in different even closely related taxa.

## Electronic supplementary material

ESM 1 (DOCX 1.01 mb)

## References

[CR1] Ahrens CW, Rymer PD, Stow A, Bragg J, Dillon S, Umbers KDL, Dudaniec RY (2018). The search for loci under selection: trends, biases and progress. Mol Ecol.

[CR2] Andrew RL, Rieseberg LH (2013). Divergence is focused on few genomic regions early in speciation: incipient speciation of sunflower ecotypes. Evolution.

[CR3] Bass A, Storey J, Dabney A, Robinson D (2015) qvalue: Q-value estimation for false discovery rate control. R package version 22.2. http://githubcom/jdstorey/qvalue

[CR4] Bogunić F, Siljak-Yakovlev S, Muratović E, Pustahija F, Medjedović S (2011). Molecular cytogenetics and flow cytometry reveal conserved genome organization in *Pinus mugo* and *P. uncinata*. Ann For Sci.

[CR5] Brennan AC, Barker D, Hiscock SJ, Abbott RJ (2012). Molecular genetic and quantitative trait divergence associated with recent homoploid hybrid speciation: a study of *Senecio squalidus* (Asteraceae). Heredity.

[CR6] Corander J, Tang J (2007). Bayesian analysis of population structure based on linked molecular information. Math Biosci.

[CR7] Critchfield WB, Little E (1966) Geographic distribution of the pines of the world. U.S. Department of Agriculture, Forest Service. Vol: 991

[CR8] Donnelly K, Cottrell J, Ennos RA, Vendramin GG, A'Hara S, King S, Perry A, Wachowiak W, Cavers S (2017). Reconstructing the plant mitochondrial genome for marker discovery: a case study using Pinus. Mol Ecol Resour.

[CR9] Excoffier L (2004). Patterns of DNA sequence diversity and genetic structure after a range expansion: lessons from the infinite-island model. Mol Ecol.

[CR10] Excoffier L, Lischer HEL (2010). Arlequin suite ver 3.5: a new series of programs to perform population genetics analyses under Linux and Windows. Mol Ecol Resour.

[CR11] Foll M, Gaggiotti O (2008). A genome-scan method to identify selected loci appropriate for both dominant and codominant markers: a Bayesian perspective. Genetics.

[CR12] González-Martinez SC, Wheeler NC, Ersoz E, Nelson CD, Neale DB (2007). Association genetics in *Pinus taeda* L. I. Wood property traits. Genetics.

[CR13] Grivet D, Sebastiani F, Alia R, Bataillon T, Torre S, Zabal-Aguirre M, Vendramin GG, Gonzalez-Martinez SC (2011). Molecular footprints of local adaptation in two mediterranean conifers. Mol Biol Evol.

[CR14] Grotkopp E, Rejmanek M, Sanderson MJ, Rost TL (2004). Evolution of genome size in pines (*Pinus*) and its life-history correlates: supertree analyses. Evolution.

[CR15] Guichoux E, Garnier-Géré P, Lagache L, Lang T, Boury C, Petit RJ (2013). Outlier loci highlight the direction of introgression in oaks. Mol Ecol.

[CR16] Heuertz M, Teufel J, González-Martínez SC, Soto A, Fady B, Alía R, Vendramin GG (2010). Geography determines genetic relationships between species of mountain pine *Pinus mugo* complex in western Europe. J Biogeogr.

[CR17] Hey J, Wakeley J (1997). A coalescent estimator of the population recombination rate. Genetics.

[CR18] Hudson RR, Kreitman M, Aguade M (1987). A test of neutral molecular evolution based on nucleotide data. Genetics.

[CR19] Ingvarsson PK, Garcia MV, Hall D, Luquez V, Jansson S (2006). Clinal variation in phyB2, a candidate gene for day-length-induced growth cessation and bud set, across a latitudinal gradient in European aspen (*Populus tremula*). Genetics.

[CR20] Jasińska AK, Wachowiak W, Muchewicz E, Boratyńska K, Montserrat JM, Boratyński A (2010). Cryptic hybrids between *Pinus uncinata* and *P. sylvestris*. Bot J Linn Soc.

[CR21] Jiggins CD, Salazar C, Linares M, Mavarez J (2008). Hybrid trait speciation and *Heliconius* butterflies. Philos Trans R Soc B.

[CR22] Lewandowski A, Boratyński A, Mejnartowicz L (2000). Allozyme investigations on the genetic differentiation between closely related pines *Pinus sylvestris*, *P. mugo*, *P. uncinata* and *P. uliginosa* (Pinaceae). Plant Syst Evol.

[CR23] Lexer C, Joseph JA, van Loo M, Barbara T, Heinze B, Bartha D, Castiglione S, Fay MF, Buerkle CA (2010). Genomic admixture analysis in European populus spp. reveals unexpected patterns of reproductive isolation and mating. Genetics.

[CR24] Librado P, Rozas J (2009). DnaSP v5: a software for comprehensive analysis of DNA polymorphism data. Bioinformatics.

[CR25] Lynch M, Crease TJ (1990). The analysis of population survey data on DNA sequence variation. Mol Biol Evol.

[CR26] Martin SH, Dasmahapatra KK, Nadeau NJ, Salazar C, Walters JR, Simpson F, Blaxter M, Manica A, Mallet J, Jiggins CD (2013). Genome-wide evidence for speciation with gene flow in *Heliconius* butterflies. Genome Res.

[CR27] Mosca E, Eckert AJ, Liechty JD, Wegrzyn JL, La Porta N, Vendramin GG, Neale DB (2012). Contrasting patterns of nucleotide diversity for four conifers of Alpine European forests. Evol Appl.

[CR28] Neale D (2014). Decoding the massive genome of loblolly pine using haploid DNA and novel assembly strategies. Genome Biol.

[CR29] Nei M (1987). Molecular evolutionary genetics.

[CR30] Nosil P, Feder JL (2013). Genome evolution and speciation: toward quantitative descriptions of pattern and process. Evolution.

[CR31] Nystedt B, Street NR, Wetterbom A, Zuccolo A, Lin YC, Scofield DG, Vezzi F, Delhomme N, Giacomello S, Alexeyenko A, Vicedomini R, Sahlin K, Sherwood E, Elfstrand M, Gramzow L, Holmberg K, Hällman J, Keech O, Klasson L, Koriabine M, Kucukoglu M, Käller M, Luthman J, Lysholm F, Niittylä T, Olson Å, Rilakovic N, Ritland C, Rosselló JA, Sena J, Svensson T, Talavera-López C, Theißen G, Tuominen H, Vanneste K, Wu ZQ, Zhang B, Zerbe P, Arvestad L, Bhalerao R, Bohlmann J, Bousquet J, Garcia Gil R, Hvidsten TR, de Jong P, MacKay J, Morgante M, Ritland K, Sundberg B, Lee Thompson S, van de Peer Y, Andersson B, Nilsson O, Ingvarsson PK, Lundeberg J, Jansson S (2013). The Norway spruce genome sequence and conifer genome evolution. Nature.

[CR32] Pritchard JK, Stephens M, Donnelly P (2000). Inference of population structure using multilocus genotype data. Genetics.

[CR33] Pyhäjärvi T, Garcia-Gil MR, Knürr T, Mikkonen M, Wachowiak W, Savolainen O (2007). Demographic history has influenced nucleotide diversity in European *Pinus sylvestris* populations. Genetics.

[CR34] Rellstab C, Zoller S, Walthert L, Lesur I, Pluess AR, Graf R, Bodénès C, Sperisen C, Kremer A, Gugerli F (2016). Signatures of local adaptation in candidate genes of oaks (*Quercus* spp.) with respect to present and future climatic conditions. Mol Ecol.

[CR35] Rieseberg LH, Raymond O, Rosenthal DM, Lai Z, Livingstone K, Nakazato T, Durphy JL, Schwarzbach AE, Donovan LA, Lexer C (2003). Major ecological transitions in wild sunflowers facilitated by hybridization. Science.

[CR36] Rundle HD, Nosil P (2005). Ecological speciation. Ecol Lett.

[CR37] Scascitelli M, Whitney KD, Randell RA, King M, Buerkle CA, Rieseberg LH (2010). Genome scan of hybridizing sunflowers from Texas (*Helianthus annuus* and *H. debilis*) reveals asymmetric patterns of introgression and small islands of genomic differentiation. Mol Ecol.

[CR38] Song BH, Wang XQ, Wang XR, Ding KY, Hong DY (2003). Cytoplasmic composition in *Pinus densata* and population establishment of the diploid hybrid pine. Mol Ecol.

[CR39] Storey JD, Tibshirani R (2003). Statistical significance for genomewide studies. Proc Natl Acad Sci U S A.

[CR40] Strasburg JL, Sherman NA, Wright KM, Moyle LC, Willis JH, Rieseberg LH (2012). What can patterns of differentiation across plant genomes tell us about adaptation and speciation?. Philos Trans R Soc B.

[CR41] Tajima F (1989). Statistical-method for testing the neutral mutation hypothesis by DNA polymorphism. Genetics.

[CR42] Turner TL, Von Wettberg EJ, Nuzhdin SV (2008). Genomic analysis of differentiation between soil types reveals candidate genes for local adaptation in *Arabidopsis lyrata*. PLoS One.

[CR43] Varis S, Pakkanen A, Galofré A, Pulkkinen P (2009). The extent of south-north pollen transfer in Finnish Scots pine. Silva Fenn.

[CR44] Wachowiak W, Prus-Głowacki W (2008). Hybridisation processes in sympatric populations of pines *Pinus sylvestris* L., *P. mugo* Turra and *P. uliginosa* Neumann. Plant Syst Evol.

[CR45] Wachowiak W, Balk P, Savolainen O (2009). Search for nucleotide diversity patterns of local adaptation in dehydrins and other cold-related candidate genes in Scots pine (*Pinus sylvestris* L.). Tree Genet Genomes.

[CR46] Wachowiak W, Palme AE, Savolainen O (2011). Speciation history of three closely related pines *Pinus mugo* (T.), *P. uliginosa* (N.) and *P. sylvestris* (L.). Mol Ecol.

[CR47] Wachowiak W, Boratyńska K, Cavers S (2013). Geographical patterns of nucleotide diversity and population differentiation in three closely related European pine species in the *Pinus mugo* complex. Bot J Linn Soc.

[CR48] Watterson G.A. (1975). On the number of segregating sites in genetical models without recombination. Theor Popul Biol.

[CR49] Yatabe Y, Kane NC, Scotti-Saintagne C, Rieseberg LH (2007). Rampant gene exchange across a strong reproductive barrier between the annual sunflowers, *Helianthus annuus* and *H. petiolaris*. Genetics.

[CR50] Yeaman S, Hodgins KA, Lotterhos KE, Suren H, Nadeau S, Degner JC, Nurkowski KA, Smets P, Wang T, Gray LK, Liepe KJ, Hamann A, Holliday JA, Whitlock MC, Rieseberg LH, Aitken SN (2016). Convergent local adaptation to climate in distantly related conifers. Science.

[CR51] Zeng K, Shi S, Wu C-I (2007). Compound tests for the detection of hitchhiking under positive selection. Mol Biol Evol.

