# Supplementary material for: Molecular signatures of divergence and selection in closely related pine taxa
Source: Tree Genet Genomes. 2018 Oct 28;14(6):83. doi: 10.1007/s11295-018-1296-3 (PMC6404648; doi:10.1007/s11295-018-1296-3)
Supplement: Supplementary file 1 — (DOCX 1.01 mb) [file 11295_2018_1296_MOESM1_ESM.docx]

**Supplementary Material**

**Molecular signatures of divergence and selection in closely related pine taxa**

**Tree Genetics & Genomes**

Witold Wachowiak^1,2,3^**^*^**, Julia Zaborowska^3^, Bartosz Łabiszak^3^, Annika Perry^1^, Giovanni M. Zucca^1^, Santiago C. González-Martínez^4^, Stephen Cavers^1^**^*^**

^1^ Centre for Ecology and Hydrology Edinburgh, Bush Estate, Penicuik, Midlothian EH26 0QB, UK

^2^ Institute of Dendrology, Polish Academy of Sciences, Parkowa 5, 62-035 Kórnik, Poland

^3^ Institute of Environmental Biology, Faculty of Biology, Adam Mickiewicz University, Umultowska 89, 61-614 Poznań, Poland

^4^ BIOGECO, INRA, Univ. Bordeaux, 33610 Cestas, France

*Corresponding authors:

E-mail: [witwac@amu.edu.pl](mailto:witwac@amu.edu.pl) (WW)

E-mail: [scav@ceh.ac.uk](mailto:witcho@ceh.ac.uk) (SC)

**Table S1.** Location of populations of the pine taxa and corresponding environmental variables. Groups of populations defined include *Pinus mugo* from Central Europe (Sudety Mts. and Alps), Carpathians (eastern and southern) and Balkans (Pirin and Durmitor Mts.). PM – *Pinus mugo*, PUG – *P. uliginosa*, PUN – *P. uncinata*

| **Acr.** | **Location** | **Longitude** | **Latitude** | **Altitude [m a.s.l.]** |
| --- | --- | --- | --- | --- |
| PM1 | Poland_Śląskie Kamienie | 15^0^36’ 8” | 50^0^46’35” | 1300 |
| PM4 | Romania_Eastern Carpathians | 24^0^48’00” | 47^0^34’03” | 1720 |
| PM5 | Romania_Southern Carpathians | 25^0^27’06” | 45^0^25’55” | 2070 |
| PM7 | Bulgaria_Pirin | 23^0^25’22” | 41^0^46’07” | 2000 |
| PM8 | Montenegro_Durmitor Mts. | 19^0^05’27” | 43^0^09’33” | 2100 |
| PM12 | Austria_Karwendel Alps | 11^0^17’45” | 47^0^22’42” | 1400 |
| PM14 | Italy_Carnic Alps | 13^0^08’ 50” | 46^0^32’40” | 1300 |
| PM16 | Italy_Abruzzi | 13^0^58’30” | 41^0^46’20” | 2200 |
| PUG1 | Poland_Węgliniec reserve | 15^0^14’20” | 51^0^17’50” | 190 |
| PUG2 | Germany_Mitelwalde | 11^0^16’27” | 47^0^28’50” | 856 |
| PUG3 | Poland_Batorów reserve | 16^0^23’1” | 50^0^27’ 32” | 710 |
| PUN17 | Andora_Vall de Ransol | 1^0^38’21” | 42^0^35’02” | 2025 |
| PUN18 | Andora_San Miguel de Engolasters | 1^0^34’12” | 42^0^31’28” | 2000 |
| PUN23 | Spain_Castiello de Jaca | -0^0^32’12” | 42^0^41’19” | 1720 |
| PUN24 | Spain_Sierra de Gudar | 0^0^41’51” | 40^0^28’49” | 2000 |
| PUN28 | France_Col de la Croix de Morano | 2^0^50’44” | 45^0^35’58” | 1400 |

**Table S2.** Analysed loci in the three pine taxa including *Pinus mugo*, *P. uliginosa and P. uncinata*.

| **Locus** | **Locus** |  |  |  | **Base pairs screened ^4^** | | | **Gene Bank Acc. Nr.^6^** |
| --- | --- | --- | --- | --- | --- | --- | --- | --- |
|  | **Acronym** | **PCR Primers (F-upper, and R-lower)** | **Ta** | **Gene function [Category^3^]** | **Tot.** | **Silent** | **Indel ^5^** |  |
| **1** | **Pr1_1** | **^1^AGGAAGGAAGGGGAAAC** |  | **putative beta-alanine ligase [M]** | **359** | **96** | **-** | **KC979156 - KC979177** |
|  |  | **^2^CAGGCTCCACTATATTG** | **60 ^0^C** |  |  |  |  |  |
| **2** | **Pr1_3** | **^1^ACATCAAGCCAGGAAG** |  | **hydrolase [M]** | **591** | **509** | **2 (8)** | **KC979178 - KC979193** |
|  |  | **^2^CCAGCAAGTGTTACAG** | **60 ^0^C** |  |  |  |  |  |
| **3** | **Pr1_5** | **^1^GATCATTCTAGGCACAGCACAAG** |  | **Seryl-tRNA synthetase [E]** | **532** | **464** | **1(5)** | **KC979194 - KC979222** |
|  |  | **^2^CCTGTACCGTGTTCATCAATTTAGCAAG** | **60 ^0^C** |  |  |  |  |  |
| **4** | **Pr1_9** | **^1^GGAATTAAAACCAGACGAAC** |  | **transcription factor [E]** | **286** | **213** | **-** | **KC979223 - KC979245** |
|  |  | **^2^GAAGAGGAATGACCAG** | **60 ^0^C** |  |  |  |  |  |
| **5** | **Pr1_10** | **^1^CAACAATGGCAAGAGAC** |  | **transcribed locus [UN]** | **361** | **221** | **-** | **KC979246 - KC979258** |
|  |  | **^2^AACGATTACCTCGCTG** | **60 ^0^C** |  |  |  |  |  |
| **6** | **Pr1_11** | **^1^GACCAGGCAAGGAAACAAAAG** |  | **putative glucuronidase 3 [M]** | **721** | **617** | **1 (7)** | **KC979259 - KC979288** |
|  |  | **^2^TTGGCAATCGGTTGATGGGGAG** | **60 ^0^C** |  |  |  |  |  |
| **7** | **Pr1_12** | **^1^TCCCCATTCTCCAAAC** |  | **NAD(P)-linked oxidoreductase-** | **442** | **279** | **1 (3)** | **KC979289 - KC979325** |
|  |  | **^2^CCATCCAATCCTTCATC** | **60 ^0^C** | **like protein [M]** |  |  |  |  |
| **8** | **Pr1_13** | **^1^AACGCTGTGACTTGTCC** |  | **hypothetical protein [UN]** | **531** | **391** | **-** | **KC979326 - KC979347** |
|  |  | **^2^CTTGTAATTCAGATATTCACGGGG** | **60 ^0^C** |  |  |  |  |  |
| **9** | **Pr1_14** | **^1^CTGTATGGCGTTCTTC** |  | **phospholipase A1-Igamma [M]** | **354** | **260** | **-** | **KC979348 - KC979373** |
|  |  | **^2^ACTGGGCGTCAAGTTTC** | **60 ^0^C** |  |  |  |  |  |
| **10** | **Pr1_15** | **^1^CATTATTATCCAAGGGCGAG** |  | **mitogen-activated protein-** | **593** | **428** | **4(29)** | **KC979374 - KC979410** |
|  |  | **^2^GAGGCTTTGAGTCACCGTTAC** | **60 ^0^C** | **kinase [ST]** |  |  |  |  |
| **11** | **Pr1_16** | **^1^GCACCCTTTGTTATGAATTGTTGTC** |  | **hypothetical protein [UN]** | **346** | **346** | **-** | **KC979411 - KC979423** |
|  |  | **^2^GGGTTTCATCAACCTCCATTCAATAGCG** | **60 ^0^C** |  |  |  |  |  |
| **12** | **Pr1_17** | **^1^TGGGTTGTCATCTGTGG** |  | **glutamate transporter [T]** | **320** | **213** | **-** | **KC979424 - KC979435** |
|  |  | **^2^TGAGTTGCTGTGAGAGG** | **60 ^0^C** |  |  |  |  |  |
| **13** | **Pr1_18** | **^1^AAGCGACTCAAAAGGGG** |  | **alpha 1,3-glucosidase [M]** | **516** | **399** | **3 (15)** | **KC979436 - KC979460** |
|  |  | **^2^TCGGCTGTATTGTCTC** | **60 ^0^C** |  |  |  |  |  |
| **14** | **Pr1_19** | **^1^CCGTATGCAAAGCATTTC** |  | **Glycosyltransferase [M]** | **366** | **366** | **-** | KC979461-KC979482 |
|  |  | **^2^ACCTGATCGTGTTGTG** | **60 ^0^C** |  |  |  |  |  |
| **15** | **Pr1_21** | **^1^GGGTGCATGTTTCATCCACAG** |  | **histone H3 K4-specific-** | **361** | **361** | **1 (9)** | KC979483-KC979505 |
|  |  | **^2^GCAGCAGCAAAAGCATTTGAAG** | **60 ^0^C** | **methyltransferase [M]** |  |  |  |  |
| **16** | **Pr1_22** | **^1^TGAAGGGAGAGGACTAC** |  | **hypothetical protein [UN]** | **301** | **225** | **-** | KC979506-KC979528 |
|  |  | **^2^ACCCAGAAACACAAAGAGGAAAC** | **60 ^0^C** |  |  |  |  |  |
| **17** | **Pr1_24** | **^1^ATGGATATTCTCCATGATGCAC** |  | **putative S-adenosylmethionine-** | **312** | **137** | **1 (10)** | KC979529-KC979537 |
|  |  | **^2^ATGGTCGTCTTTTTGCTTC** | **60 ^0^C** | **dependent protein [ST]** |  |  |  |  |
| **18** | **Pr1_26** | **^1^CCCATTTTAGCAAACCC** |  | **putative pre-mRNA branch site-** | **409** | **109** | **3 (5)** | **KC979538 - KC979603** |
|  |  | **^2^GAAGTGAAGATGAGCATAAG** | **60 ^0^C** | **protein p14 [E]** |  |  |  |  |
| **19** | **Pr1_28** | **^1^GCAACTTCCCCTTTTTC** |  | **translation initiation factor-** | **522** | **424** | **-** | **KC979604 - KC979621** |
|  |  | **^2^ACAGTGTGAGAGACGAG** | **60 ^0^C** | **4G-like [E]** |  |  |  |  |
| **20** | **Pr1_29** | **^1^CCATTGGTGTGTCTTCTC** |  | **short-chain dehydrogenase** | **279** | **180** | **-** | **KC979622 - KC979653** |
|  |  | **^2^AATACCCTTTCAAGGCAAGCATATC** | **60 ^0^C** | **Protein [M]** |  |  |  |  |
| **21** | **Pr1_31** | **^1^TGTGAAGCAGAGGAAC** |  | **putative patellin-4 [T]** | **315** | **91** | **2 (6)** | **KC979654 - KC979673** |
|  |  | **^2^GATGGTAAGGATGGTG** | **60 ^0^C** |  |  |  |  |  |
| **22** | **Pr1_36** | **^1^GCGTTCATCATCTCAAGCC** |  | **transcribed locus [UN]** | **434** | **224** | **3 (21)** | **KC979674 - KC979696** |
|  |  | **^2^CTAATCTCTCTTATTGTCATCTCCACC** | **60 ^0^C** |  |  |  |  |  |
| **23** | **Pr1_40** | **^1^TACAATATGTTCCTGTCAAGGC** |  | **Homeobox domain containing -** | **489** | **257** | **1 (1)** | **KC979697 - KC979721** |
|  |  | **^2^TACGCTCACATGGTCTCCTTC** | **60 ^0^C** | **Protein [ST]** |  |  |  |  |
| **24** | **Pr1_43** | **^1^GGACATTGTACTGTTGG** |  | **beta-galactosidase [M]** | **591** | **375** | **-** | **KC979722 - KC979747** |
|  |  | **^2^GGGTAAATGGAAAGAGTATTGG** | **60 ^0^C** |  |  |  |  |  |
| **25** | **Pr1_45** | **^1^GAAATAGTCCTCTTCCTTTG** |  | **O-fucosyltransferase-like protein** | **341** | **341** | **1 (3)** | **KC979748 - KC979765** |
|  |  | **^2^GGCTGCTTTGGATTATATTG** | **60 ^0^C** | **[M]** |  |  |  |  |
| **26** | **Pr1_46** | **^1^ATCCAGTCCCTTCTCCACCTATCC** |  | **alpha-N-acetylglucosaminidase[M]** | **511** | **437** | **2 (10)** | **KC979766 - KC979785** |
|  |  | **^2^AAGTGCTAAGCATCAAGCAGATAATCC** | **60 ^0^C** |  |  |  |  |  |
| **27** | **Pr1_47** | **^1^GTAAATCTTCTTGCCTCTTCATCC** |  | **glycoprotein** | **558** | **451** | **2 (16)** | **KC979786 - KC979800** |
|  |  | **^2^TATGCTCAACATACAGTACC** | **60 ^0^C** | **glucosyltransferase[M]** |  |  |  |  |
| **28** | **Pr1_48** | **^1^ACCAATGCACATGCCAC** |  | **transport protein [T]** | **384** | **344** | **1 (1)** | KC979801- KC979827 |
|  |  | **^2^TATTACATCACTCCACCTTC** | **60 ^0^C** |  |  |  |  |  |
| **29** | **Pr2_3** | **^1^GAGGAGATGGTTTTGTATG** |  | **2-3 ethylene-responsive-** | **365** | **98** | **-** | **KC979828 - KC979847** |
|  |  | **^2^ATCCAGGTGCTACTTC** | **60 ^0^C** | **transcription factor 1B-like [E]** |  |  |  |  |
| **30** | **Pr2_4** | **^1^GACTGGGATACTCTTTTGG** |  | **f-box family protein [ST]** | **410** | **113** | **-** | **KC979848 - KC979854** |
|  |  | **^2^AGTGAATAACACTTGGCTGCTATAC** | **60 ^0^C** |  |  |  |  |  |
| **31** | **Pr2_5** | **^1^ATTCATCCACTTCCCCC** |  | **transcribed locus [UN]** | **299** | **81** | **1 (3)** | **KC979855 - KC979874** |
|  |  | **^2^GTGTATTTGGTGTTGCAG** | **60 ^0^C** |  |  |  |  |  |
| **32** | **Pr2_7** | **^1^CAAAACCCTTTGAGCAC** |  | **transcribed locus [UN]** | **432** | **131** | **-** | **KC979875 - KC979887** |
|  |  | **^2^GAGAACTTCTTCCATTCC** | **60 ^0^C** |  |  |  |  |  |
| **33** | **Pr2_9** | **^1^CCTCAAGGGACATGAAAATATTGAC** |  | **hypothetical protein [UN]** | **381** | **296** | **1 (1)** | **KC979888 - KC979903** |
|  |  | **^2^AAAGGCGATAAGGAAAAGGCAC** | **60 ^0^C** |  |  |  |  |  |
| **34** | **Pr2_11** | **^1^ACAGCAGCGATTCAAC** |  | **3-ketoacyl-CoA synthase 6-like[M]** | **299** | **151** | **2 (13)** | **KC979904 - KC979919** |
|  |  | **^2^AACACCTCTTCCTCGTC** | **60 ^0^C** |  |  |  |  |  |
| **35** | **Pr2_12** | **^1^GTAAGCTATATCCAGAACC** |  | **protein kinase [ST]** | **342** | **88** | **-** | **KC979920 - KC979935** |
|  |  | **^2^CGCACCTCCATCATTTTCC** | **60 ^0^C** |  |  |  |  |  |
| **36** | **Pr2_16** | **^1^TCACTTGGCAGAAGAC** |  | **glutamyl-tRNA reductase [M]** | **403** | **144** | **-** | **KC979936 - KC979946** |
|  |  | **^2^GAGAGATTCTTTGGAGAC** | **60 ^0^C** |  |  |  |  |  |
| **37** | **Pr2_17** | **^1^GCATTAGTCTGTCTGTTC** |  | **putative polyol transporter [T]** | **390** | **221** | **1 (1)** | **KC979947 - KC979961** |
|  |  | **^2^GTGTTTCTAGGGCAATC** | **60 ^0^C** |  |  |  |  |  |
| **38** | **Pr2_20** | **^1^TCGAAGACAAGCTCTG** |  | **GDP-dissociation inhibitor family-** | **379** | **245** | **1 (6)** | **KC979962 - KC979984** |
|  |  | **^2^GACGACGATAAATGCTAC** | **60 ^0^C** | **Protein [T]** |  |  |  |  |
| **39** | **Pr2_23** | **^1^GCCCAAATGGTTATACATAACACTC** |  | **Transcribed locus [UN]** | **379** | **115** | **-** | **KC979985 - KC980011** |
|  |  | **^2^CCATTCCATCGGCACAGTCATC** | **60 ^0^C** |  |  |  |  |  |
| **40** | **Pr2_25** | **^1^TCCCTGAAATCAAATCCCAC** |  | **hypothetical protein [UN]** | **403** | **97** | **-** | **KC980012 - KC980024** |
|  |  | **^2^AACCCAGCAATCTGAGCAAAGAAAAAC** | **60 ^0^C** |  |  |  |  |  |
| **41** | **Pr2_28** | **^1^CCTCCCATCATTCTTTCTTCC** |  | **basic leucine zipper** | **484** | **229** | **-** | **KC980025 - KC980041** |
|  |  | **^2^GAATTGCAGCCCTTGCACAAGAC** | **60 ^0^C** | **transcription-factor-like protein [E]** |  |  |  |  |
| **42** | **Pr2_29** | **^1^GGCTTTGAACACCCTCAAAAATAC** |  | **hypothetical protein [UN]** | **374** | **198** | **2 (4)** | **KC980042 - KC980058** |
|  |  | **^2^TAAGGACATCAATACCAGTTTGCTCAG** | **60 ^0^C** |  |  |  |  |  |
| **43** | **Pr2_30** | **^1^CACTTGTCATCTGCTC** |  | **U-box domain-containing [ST]** | **402** | **98** | **-** | **KC980059 - KC980073** |
|  |  | **^2^CTTGGAAGGATAGAATCTG** | **60 ^0^C** | **protein** |  |  |  |  |
| **44** | **Pr2_32** | **^1^GAATAGAAATAGAGTGCGATGG** |  | **hypothetical protein [UN]** | **378** | **378** | **-** | **KC980074 - KC980091** |
|  |  | **^2^AAAAATGATGGCTGCGTGGAGG** | **60 ^0^C** |  |  |  |  |  |
| **45** | **Pr2_34** | **^1^CATTTCCAAGAGAAGACGAC** |  | **ATP/ADP transporter [T]** | **375** | **90** | **1 (9)** | **KC980092 - KC980103** |
|  |  | **^2^TTGCCAACTCCACTCCCTAC** | **60 ^0^C** |  |  |  |  |  |
| **46** | **Pr2_35** | **^1^ACCCACAAATTGCCAG** |  | **DEAD-box ATP-dependent** | **420** | **291** | **1 (3)** | **KC980104 - KC980122** |
|  |  | **^2^GCCGTGATTATCGAAGAG** | **60 ^0^C** | **RNA- helicase [M]** |  |  |  |  |
| **47** | **Pr2_38** | **^1^CCATCATACAACTATCCAC** |  | **hypothetical protein [UN]** | **451** | **260** | **-** | **KC980123 - KC980143** |
|  |  | **^2^ACAGAGAATAATGGGGCAC** | **60 ^0^C** |  |  |  |  |  |
| **48** | **Pr2_41** | **^1^GAAAAGGATCAAATTGTGGG** |  | **F-box protein GID2 [ST]** | **378** | **285** | **-** | **KC980144 - KC980160** |
|  |  | **^2^GCTAACATTGGCTGTGG** | **60 ^0^C** |  |  |  |  |  |
| **49** | **Pr2_42** | **^1^GCATAGCCATCCATATC** |  | **transducin/WD40 domain-** | **511** | **381** | **-** | **KC980161 - KC980175** |
|  |  | **^2^GGGTGTGAATTTTTTTGGTG** | **60 ^0^C** | **containing protein [ST]** |  |  |  |  |
| **50** | **Pr2_44** | **^1^AGCACCAGACATAGCAC** |  | **DNA repair helicase XPB1-like[M]** | **290** | **290** | **1 (19)** | **KC980176 - KC980188** |
|  |  | **^2^TCATTACAAGTTTACCGCCTC** | **60 ^0^C** |  |  |  |  |  |
| **51** | **Pr2_45** | **^1^AACCGTCCTGATGAGCCTTG** |  | **hypothetical protein [UN]** | **399** | **217** | **2 (49)** | **KC980189 - KC980218** |
|  |  | **^2^CAGCCTTTCTTACAGACAC** | **60 ^0^C** |  |  |  |  |  |
| **52** | **Pr2_47** | **^1^TTCATAAAGCCCCCCATCC** |  | **hexokinase 1 [ST]** | **549** | **549** | **3 (3)** | **KC980219 - KC980234** |
|  |  | **^2^TCTGATTTCAAAGTCGCC** | **60 ^0^C** |  |  |  |  |  |
| **53** | **Pr2_48** | **^1^GCTATGCGTTACTTGG** |  | **S-methyl-5-thioribose kinase [ST]** | **709** | **509** | **7 (22)** | **KC980235 - KC980278** |
|  |  | **^2^TGAGTTGAGCTGCTTG** | **60 ^0^C** |  |  |  |  |  |
| **54** | **175** | **CACAAAATACCAGAAAACAAATAAC** |  | **Unnamed gene ^a^ [UN]** | **431** | **191** | **-** | **KC980330 - KC980356** |
|  |  | **CGATAGAACAAAATAAAGAGGATG** | **60 ^0^C** |  |  |  |  |  |
| **55** | **a3ip** | **CGGAGTATGATGAAGAGGAACTGT** |  | **ABI3-interacting protein 2 ^b^ [ST]** | **297** | **297** | **-** | **KC980279 - KC980290** |
|  |  | **TTTCTAGCTGAGCTACACGCTTTT** | **60 ^0^C** |  |  |  |  |  |
| **56** | **abaR** | **AAGATTGCAATGATGAGTAAAGAAG** |  | **abscisic acid - responsive** | **350** | **125** | **4 (22)** | **KC980291 - KC980329** |
|  |  | **CTTGGCCCATTTGTTGAAGCAGTGA** | **60 ^0^C** | **protein ^c^ [ST]** |  |  |  |  |
| **57** | **ccoaomt** | **GCAGCAGAAGTGAAGGCTCAGA** |  | **Caffeoyl CoA O-** | **381** | **276** | **-** | **KC980357 - KC980372** |
|  |  | **TCTTTCCATCATCGGGCAATG** | **60 ^0^C** | **methyltransferase ^d^ [M]** |  |  |  |  |
| **58** | **chcs** | **ACTCCCCCTAATGCGGTTGA** |  | **chalcone synthase ^b^ [M]** | **306** | **254** | **1 (1)** | **KC980373 - KC980424** |
|  |  | **CTTGGCTGCGGCTTCTTTC** | **60 ^0^C** |  |  |  |  |  |
| **59** | **dhn2PP** | **CTGCAGAGACTGTGCCTGAGC** |  | **dehydrin ^d^ [M]** | **426** | **170** | **2 (12)** | **KC980425 - KC980465** |
|  |  | **CCAGGGAGCTTTTCCTTGATCT** | **60 ^0^C** |  |  |  |  |  |
| **60** | **eph** | **CCATGGTTCACTGAGGATGAT** |  | **putative epoxide hydrolase ^a^ [M]** | **635** | **361** | **2 (3)** | **KC980466 - KC980496** |
|  |  | **AGTTTCAAGGTCTTCCAAAAATAAG** | **60 ^0^C** |  |  |  |  |  |
| **61** | **phy** | **GCGCTGCTCTTCTCTGCTGTGCGAT** |  | **putative phytocyanin ^a^ [T]** | **463** | **283** | **1 (2)** | **KC980543 - KC980586** |
|  |  | **CCGGGCCTGTCTCGTATTTTTCC** | **60 ^0^C** |  |  |  |  |  |
| **62** | **PhytP** | **TGCAGGGCATAGACGAACTTAGC** |  | **phytochrome P ^b^ [E]** | **715** | **383** | **4 (86)** | **KC980587 - KC980605** |
|  |  | **GGCAATCTGCAAGAAACAAAACGAC** | **60 ^0^C** |  |  |  |  |  |
| **63** | **rps4** | **AGGCCGTAACAGAGGTCGTGT** |  | **putative to ribosomal** | **244** | **78** | **1 (1)** | **KC980497 - KC980506** |
|  |  | **GTTTTCCCAACAAAGTTTCCGATAA** | **60 ^0^C** | **protein S4 ^a^ [M]** |  |  |  |  |
| **64** | **rps10** | **CACCCAGAAATTGATGTTCCAAATC** |  | **ribosomal protein S10 ^a^ [M]** | **355** | **164** | **-** | **KC980507 - KC980518** |
|  |  | **CCAGCCTTRTCACCAAATTCTCCAG** | **60 ^0^C** |  |  |  |  |  |
| **65** | **hp927** | **^1^GCAATGAGGGATTGAATTAC** |  | **hypothetical protein [UN]** | **370** | **210** | **-** | **KC980519 - KC980542** |
|  |  | **^2^TTGGAAGAATACAAGGCAGG** | **60 ^0^C** |  |  |  |  |  |
| **66** | **Pr4-1** | **^1^CCGATGCTGACTCTTCTAAC** |  | **Peroxidase ^e^ [ST]** | **341** | **341** | **11(33)** | **KC980606 - KC980644** |
|  |  | **^2^AGCGAATTTGGAGGATGA** | **60 ^0^C** |  |  |  |  |  |
| **67** | **Pr4-4** | **^1^TGTCACTGCCCAGAGCTATTC** |  | **Putative aquaporin ^e^ [T]** | **640** | **496** | **7 (77)** | **KC980645 - KC980679** |
|  |  | **^2^ATCACAGCCGCTCCAAAAC** | **60 ^0^C** |  |  |  |  |  |
| **68** | **Pr4-5** | **^1^CATCTCCTTCAAACCTCTTATTTCC** |  | **calcium dependent** | **367** | **228** | **2 (10)** | **KC980680 - KC980695** |
|  |  | **^2^GATGCTTGAACATGATCCC** | **60 ^0^C** | **proteokinase ^e^ [ST]** |  |  |  |  |
| **69** | **Pr4-10** | **^1^CATTGCCTACGATTTCC** |  | **mys transcription factor ^e^ [E]** | **361** | **240** | **1 (3)** | **KC980696 - KC980702** |
|  |  | **^2^CTTTTGAGATGAACCAGAC** | **60 ^0^C** |  |  |  |  |  |
| **70** | **Pr4-11** | **^1^CCTTCTATTTGAATCCCTTG** |  | **scl1 protein ^e^ [E]** | **439** | **158** | **1 (18)** | **KC980703 - KC980724** |
|  |  | **^2^CATAGTAACAGCCTACAG** | **60 ^0^C** |  |  |  |  |  |
| **71** | **Pr4-12** | **^1^CTGCTCAAGTGAAAGG** |  | **proton myo-inositol transporter ^e^ [T]** | **535** | **419** | **1 (1)** | **KC980725 - KC980745** |
|  |  | **^2^CTGATTGTGGATTCTGTG** | **60 ^0^C** |  |  |  |  |  |
| **72** | **Pr4-17** | **^1^CTGGAAGCTGATTCTTTTG** |  | **cytochrome P450 reductase ^e^  [ST]** | **371** | **148** | **-** | **KC980746 - KC980762** |
|  |  | **^2^CCTCTAGTTCTGGTTG** | **60 ^0^C** |  |  |  |  |  |
| **73** | **Pr4-18** | **^1^AGAGAGGGAATTGGTTGAG** |  | **myblike DNA-binding protein ^e^ [E]** | **328** | **134** | **-** | **KC980763 - KC980769** |
|  |  | **^2^AAGGAAAGAAAAAGTCTGCTGATGG** | **60 ^0^C** |  |  |  |  |  |
| **74** | **Pr4-19** | **^1^CTCTACCACATCATCTCC** |  | **laccase ^e^ [M]** | **333** | **234** | **-** | **KC980770 - KC980785** |
|  |  | **^2^TTTCACTCTCGTGTCTTTCACC** | **60 ^0^C** |  |  |  |  |  |
| **75** | **Pr4-21** | **^1^ACATGGTGTTTGGCAGG** |  | **Receptor protein kinase ^e^ [ST]** | **394** | **394** | **1 (2)** | **KC980786 - KC980810** |
|  |  | **^2^AATGAGGAGGGTGGTAGAG** | **60 ^0^C** |  |  |  |  |  |
| **76** | **Pr4-27** | **^1^TAGCAGACGGTATTCACACAGTCC** |  | **putative auxin induced -** | **403** | **347** | **-** | **KC980811 - KC980829** |
|  |  | **^2^CCACAACCACCTTGCATCATTATTT** | **60 ^0^C** | **transcription factor ^e^ [E]** |  |  |  |  |
| **77** | **Pr4-34** | **^1^ACCCTGTATCGATGGGTATGGAGAT** |  | **transcription factor bHLH62-** | **360** | **285** | **1 (3)** | **KC980830 - KC980841** |
|  |  | **^2^TTTCATGTGGTTTGTTGGTACAGAACCTGCAATCA** | **60 ^0^C** | **like- gene ^e^ [E]** |  |  |  |  |
| **78** | **Pr4-38** | **^1^TTATTTACATCCAACAGCGCCATTT** |  | **SET-B-like gene ^e^ [M]** | **512** | **463** | **3 (11)** | **KC980842 - KC980863** |
|  |  | **^2^GAAAGTATGGATTGCCAACTTGCAC** | **60 ^0^C** |  |  |  |  |  |
| **79** | **Pr4-41** | **^1^TGCAAGCTGTAAGGTAAAACCCTCAT** |  | **ethylene responsive element-** | **591** | **388** | **-** | **KC980864 - KC980906** |
|  |  | **^2^CAACATCAAAACTGAAACCACCAGTC** | **60 ^0^C** | **binding protein-like gene ^e^ [ST]** |  |  |  |  |
| **80** | **Pr11** | GATCGGGTCGGAGGCATAAT  AGTTGAAGCAAGCCAGCAAG | **57 ^0^C** | **unnamed mitochondrial DNA region** ^f^ |  |  | **-** | **S6 Table** |
| **81** | **Pr34** | GAACCCCCTCTTGCCTTGAT  TTCGTGACGGTCCAATTCCA | **57 ^0^C** | **unnamed mitochondrial DNA region** |  |  | **-** | **S6 Table** |
| **82** | **Pr37** | ACACTTCCTACACGGTTCCA  TCGTTACAGAAACCCCTCGG | **57 ^0^C** | **unnamed mitochondrial DNA region** |  |  | **-** | **S6 Table** |

^1,2^ - vector sequence (1=GTAAAACGACGGCCAGT and 2=CAGGAAACAGCTATGACC) was present as a part of PCR primers used for amplification of the loci studied; Ta - annealing temperature; ^3^ – E-gene expression regulation; M-metabolisms; ST-signal transduction; T-transport; UN-unknown; ^4^-average across all samples, ^5^-number of indels and total length in parenthesis; ^6^ – Scots pine accessions were not used in the present study. DNA regions described in: ^a^ Palmé et al. (2008); ^b^ Pyhäjärvi et al. (2007); ^c^ Wachowiak et al. (2009); ^d^ Eveno et al. (2008); ^e^ Ersoz et al. (2010); ^f^  Donnelly et al. (2017).

**Table S3. A.** Net divergence per base pair at nuclear genes between the three pine taxa and silent divergence to the outgroup *Pinus taeda*. Groups defined for *P. mugo*: PMCE-Central Europe, PMCP-Carpathians, PMB-Balkans.

| **A. Group** | ***P. mugo*** | ***P. uliginosa*** | ***P. uncinata*** | **B. Group** | **PMCE** | **PMCP** | **PMB** |
| --- | --- | --- | --- | --- | --- | --- | --- |
| *P. uliginosa* | 0.0004 |  |  | PMCP | 0.0002 |  |  |
| *P. uncinata* | 0.0008 | 0.0004 |  | PMB | 0.0001 | 0.0001 |  |
| *P. taeda* | 0.0236 | 0.0234 | 0.0235 | *P. taeda* | 0.0190 | 0.0193 | 0.0191 |

**Table. S4** Percentage (%) of shared SNPs in pairwise comparisons between the three pine taxa (A) and regional groups (B) at 79 nuclear loci. Groups defined for *Pinus mugo*: PMCE-Central Europe, PMCP-Carpathians, PMB-Balkans. In parentheses: % of SNPs in taxon or groups marked in rows and columns, respectively.

| **A. Taxa** | ***P. mugo*** | ***P. uliginosa*** | **A. Group** | **PMCE** | **PMCP** |
| --- | --- | --- | --- | --- | --- |
| *P. uliginosa* | 63 (52, 80) |  | PMCP | 61 (55, 68) |  |
| *P. uncinata* | 59 (53, 65) | 69 (77, 61) | PMB | 64 (59, 70) | 67 (69, 66) |

**Table S5**. Shared and unique haplotypes between three pine taxa (A) and geographical locations (B) defined for *Pinus mugo*. Percentage of exclusive haplotypes (appearing only once) (C) as compared to the total number of haplotypes found in the taxon or corresponding regional groups.

| **A.** | ***P. mugo* (PM) vs.**  ***P. uliginosa* (PUG)** | | | ***P. mugo* (PM) vs.**  ***P. uncinata* (PUN)** | | | ***P. uliginosa* (PUG) vs.**  ***P. uncinata* (PUN)** | | | **B.** | **PMCE vs. PMCP** | | | **PMCE vs. PMB** | | | **PMCP vs PMB** | | |
| --- | --- | --- | --- | --- | --- | --- | --- | --- | --- | --- | --- | --- | --- | --- | --- | --- | --- | --- | --- |
|  | unique PM | unique PUG | shared | unique PM | unique PUN | shared | unique PUG | unique PUN | shared | unique PMCE | | unique PMCP | shared | unique PMCE | unique PMB | shared | unique PMCP | unique PMB | shared |
| **Total** | 473 | 133 | 320 | 475 | 272 | 318 | 162 | 299 | 291 | 248 | | 157 | 225 | 222 | 137 | 251 | 148 | 154 | 234 |
| **%** | 59.6 | 29.4 | 51.4 | 59.9 | 46.1 | 46.0 | 35.8 | 50.7 | 55.8 | 52.4 | | 41.1 | 52.6 | 46.9 | 35.3 | 58.3 | 38.7 | 39.7 | 60.8 |

| **C.** | ***P. mugo*** | ***P. uliginosa*** | ***P. uncinata*** | **PMCE** | **PMCP** | **PMB** |
| --- | --- | --- | --- | --- | --- | --- |
| **%** | **30.6** | **12.1** | **16.6** | **17.5** | **18.1** | **15.7** |

**Table S6.** Haplotypes detected at the *mt*DNA regions sequenced characterized in the three pine taxa. One region( Pr34) was found to be monomorphic in all surveyed taxa. SNP positions in reference to GenBank accession numbers FRDG01000215 (Pr13), APFE031732192 (Pr37). PM – *Pinus mugo*, PUG – *P. uliginosa*, PUN – *P. uncinata*.

| **No.** | **Pr11** | | | | | **Pr37** | | | | | | | | |  | | |
| --- | --- | --- | --- | --- | --- | --- | --- | --- | --- | --- | --- | --- | --- | --- | --- | --- | --- |
|  | **SNP position** | | | | | | | | | | | | | | **Frequency [% (N of samples)]** | | |
|  | **3212** | **3250** | **3299** | **3300** |  | **19357** | **19345** | **19332** | **19324** | **19312** | **19273** | **19262** | **19222** | **19165** | **PM** | **PUG** | **PUN** |
| **H1** | C | C | T | A |  | G | C | A | T | G | A | C | A | A | 8.33 (11) | 1.52 (2) | 0 (0) |
| **H2** | C | C | T | A |  | T | C | A | T | G | A | C | A | A | 31.82 (42) | 9.85 (13) | 0.76 (1) |
| **H3** | C | C | T | A |  | T | C | A | T | C | A | C | C | T | 3.79 (5) | 0 (0) | 0 (0) |
| **H4** | C | C | G | A |  | T | C | A | T | G | A | C | A | A | 0.76 (1) | 0 (0) | 0 (0) |
| **H5** | C | C | G | A |  | G | C | A | T | G | A | C | A | A | 0.76 (1) | 0 (0) | 0 (0) |
| **H6** | C | C | T | A |  | T | C | A | C | G | A | C | A | A | 2.27 (3) | 0.76 (1) | 0.76 (1) |
| **H7** | C | C | T | A |  | T | C | A | T | G | G | G | A | A | 1.52 (2) | 1.52 (2) | 0 (0) |
| **H8** | C | C | T | A |  | T | G | A | T | G | A | C | A | A | 0.76 (1) | 0 (0) | 0 (0) |
| **H9** | C | C | T | A |  | T | C | A | T | G | A | C | C | A | 0 (0) | 1.52 (2) | 0 (0) |
| **H10** | C | C | T | A |  | T | C | A | T | C | A | C | A | T | 0 (0) | 0.76 (1) | 0 (0) |
| **H11** | C | A | T | A |  | T | C | A | T | G | A | C | A | A | 0 (0) | 0 (0) | 20.45 (27) |
| **H12** | C | A | T | A |  | T | C | A | T | C | A | C | C | T | 0 (0) | 0 (0) | 1.52 (2) |
| **H13** | C | A | T | C |  | G | C | A | T | G | A | C | A | A | 0 (0) | 0 (0) | 0.76 (1) |
| **H14** | C | A | T | C |  | T | C | A | T | G | A | C | A | A | 0 (0) | 0 (0) | 0.76 (1) |
| **H15** | C | A | T | C |  | T | C | A | T | G | G | G | A | A | 0 (0) | 0 (0) | 1.52 (2) |
| **H16** | C | A | T | A |  | T | C | A | T | G | G | G | A | A | 0 (0) | 0 (0) | 4.55 (6) |
| **H17** | A | A | T | A |  | T | C | A | T | G | A | C | A | A | 0 (0) | 0 (0) | 0.76 (1) |
| **H18** | A | A | T | A |  | G | C | A | T | G | A | C | A | A | 0 (0) | 0 (0) | 0.76 (1) |
| **H19** | C | A | T | A |  | T | C | G | T | G | A | C | A | A | 0 (0) | 0 (0) | 0.76 (1) |
| **H20** | C | A | T | A |  | T | C | A | T | G | A | C | C | A | 0 (0) | 0 (0) | 0.76 (1) |
|  |  |  |  |  |  |  |  |  |  |  |  |  |  |  | **50 (61)** | **15.85(21)** | **34.12 (50)** |

**Table S7.** Global AMOVA as weighted average over all SNPs.

| Source of variation | Sum of squares | Variance components | Percentage variation |
| --- | --- | --- | --- |
| ***Pinus mugo* complex (3 taxa) – 1,212 SNPs** | | | |
| Among groups | 1031.7 | 9.4 | 11.4 |
| Among populations within groups | 1335.0 | 3.6 | 4.4 |
| Within populations | 9018.0 | 69.3 | 84.2 |
| Total |  |  |  |
| ***P. mugo -* 892 SNPs** | | | |
| Among populations | 680.7 | 3.5 | 5.3 |
| Within populations | 4290.0 | 63.8 | 94.7 |
| Total | 4970.7 | 67.4 |  |
| ***P. mugo* three regional groups - 844 SNPs** | | | |
| Among regions | 201.9 | 1.7 | 2.5 |
| Within regions | 4092.8 | 65.1 | 97.5 |
| Total | 4294.8 | 66.7 |  |
| ***P. uliginosa -* 595 SNPs** | | | |
| Among populations | 228.2 | 5.4 | 6.9 |
| Within populations | 1457.1 | 73.8 | 93.1 |
| Total | 1685.3 | 79.2 |  |
| ***P. uncinata* - 739 SNPs** | | | |
| Among populations | 426.8 | 3.2 | 4.0 |
| Within populations | 3279.5 | 76.1 | 96.0 |
| Total | 3706.3 | 79.3 |  |

**Table S8.** Genes that showed deviations from neutral expectations in compound neutrality (HEW and/or DHEW) tests across the taxa (p<0.05).

| ***Acronym*** | **Gene** | ***Pinus mugo*** | ***P. uliginosa*** | ***P. uncinata*** |
| --- | --- | --- | --- | --- |
| Pr1_5 | **seryl-tRNA synthetase [E]** | DHEW | HEW, DHEW | HEW |
| Pr1_9 | **transcription factor [E]** |  | HEW, DHEW |  |
| Pr1_11 | **putative glucuronidase 3 [M]** | HEW, DHEW |  |  |
| Pr1_15 | **mitogen-activated protein- kinase [ST]** | HEW, DHEW | HEW, DHEW | HEW, DHEW |
| Pr1_16 | **hypothetical protein [UN]** | HEW, DHEW | HEW, DHEW |  |
| Pr1_28 | **glutamate transporter [T]** | HEW | HEW |  |
| Pr1_40 | **homeobox domain containing - Protein [ST]** | HEW, DHEW |  |  |
| Pr1_46 | **alpha-N-acetylglucosaminidase[M]** | HEW, DHEW | HEW |  |
| Pr1_48 | **transport protein [T]** |  |  | HEW, DHEW |
| Pr2_3 | **2-3 ethylene-responsive- transcription factor 1B-like [E]** |  | HEW |  |
| Pr2_5 | **transcribed locus [UN]** | HEW, DHEW |  |  |
| Pr2_7 | **transcribed locus [UN]** |  |  | HEW, DHEW |
| Pr2_11 | **3-ketoacyl-CoA synthase 6-like[M]** | HEW, DHEW |  |  |
| Pr2_20 | **GDP-dissociation inhibitor family- Protein [T]** | DHEW | HEW, DHEW |  |
| Pr2_28 | **basic leucine zipper transcription-factor-like protein [E]** | HEW, DHEW |  |  |
| Pr2_34 | **ATP/ADP transporter [T]** |  | HEW, DHEW |  |
| Pr2_42 | **transducin/WD40 domain- containing protein [ST]** | HEW |  |  |
| Pr2_44 | **DNA repair helicase XPB1-like[M]** |  | HEW |  |
| 175 | **unnamed gene ^a^ [UN]** | HEW, DHEW |  |  |
| abaR | **abscisic acid - responsive protein ^c^ [ST]** |  |  | HEW |
| ccoam | **Caffeoyl CoA O- methyltransferase ^d^ [M]** | HEW, DHEW |  | HEW, DHEW |
| phy | **putative phytocyanin ^a^ [T]** | DHEW |  |  |
| phytP | **phytochrome P ^b^ [E]** | HEW, DHEW |  |  |
| rps10 | **ribosomal protein S10 ^a^ [M]** | HEW |  |  |
| Pr4-1 | **Peroxidase ^e^ [ST]** |  | DHEW | HEW |
| Pr4-5 | **calcium dependent proteokinase ^e^ [ST]** | HEW, DHEW | HEW |  |
| Pr4-27 | **putative auxin induced - transcription factor ^e^ [E]** | HEW, DHEW | HEW, DHEW |  |

**Table S9.** Probability from chi square distribution for correlation between polymorphism and divergence at the analysed taxa and as compared to the outgroup *Pinus taeda* using the HKA test (Hudson et al. 1987).

| **Taxa** | ***Pinus mugo*** | ***P. uliginosa*** | ***P. uncinata*** |
| --- | --- | --- | --- |
| *P. uliginosa* | 0.99 |  |  |
| *P. uncinata* | 0.98 | 0.99 |  |
| *P. taeda* | 0.07 | 0.89 | 0.16 |

**Table S10.** Pairwise Fst values between analysed taxa and groups of populations defined for *Pinus mugo* at 79 gene fragments. Statistically significant Fst values (p<0.01) are marked in bold. q value: false positive rates estimate to correct for multiple testing (q<0.05 is marked in red).

| **Gene** | **PM-PUG** | **q value** | **PM-PUN** | **q value** | **PUG-PUN** | **q value** | **PMCE-PMCP** | **q value** | **PMCE-PMB** | **q value** | **PMCP-PMB** | **q**  **value** | **PUG** | **q**  **value** | **PUN** | **q**  **value** |
| --- | --- | --- | --- | --- | --- | --- | --- | --- | --- | --- | --- | --- | --- | --- | --- | --- |
| **Pr1_1** | -0.001 | 0.3664 | 0.022 | 0.0390 | **0.133** | 0.0000 | 0.079 | 0.2111 | 0.018 | 0.5669 | -0.033 | 0.8090 | 0.135 | 0.2520 | 0.027 | 0.4721 |
| **Pr1_3** | 0.039 | 0.3270 | -0.007 | 0.2350 | -0.010 | 0.2393 | 0.017 | 0.5039 | -0.037 | 0.8571 | 0.052 | 0.5255 | 0.071 | 0.3662 | 0.067 | 0.4202 |
| **Pr1_5** | -0.018 | 0.5874 | 0.006 | 0.1455 | 0.025 | 0.1653 | 0.020 | 0.3780 | 0.014 | 0.7034 | 0.016 | 0.5912 | -0.035 | 0.6049 | 0.001 | 0.5271 |
| **Pr1_9** | -0.013 | 0.4672 | 0.000 | 0.2842 | -0.011 | 0.3103 | -0.006 | 0.5558 | -0.004 | 0.7034 | -0.020 | 0.8773 | -0.005 | 0.4958 | 0.018 | 0.4721 |
| **Pr1_10** | -0.020 | 0.6093 | 0.040 | 0.0240 | **0.099** | 0.0000 | 0.075 | 0.1040 | 0.054 | 0.4873 | -0.030 | 0.8926 | -0.105 | 0.7915 | 0.040 | 0.4450 |
| **Pr1_11** | -0.015 | 0.4604 | **0.027** | 0.0129 | 0.067 | 0.0494 | -0.023 | 0.5039 | -0.024 | 0.7034 | -0.050 | 0.8090 | -0.122 | 0.7915 | 0.003 | 0.5006 |
| **Pr1_12** | -0.003 | 0.4207 | 0.015 | 0.1324 | 0.059 | 0.0682 | -0.023 | 0.6175 | 0.033 | 0.5669 | -0.012 | 0.8050 | **0.249** | 0.1440 | 0.054 | 0.3988 |
| **Pr1_13** | -0.002 | 0.4604 | 0.021 | 0.0390 | 0.076 | 0.0309 | 0.048 | 0.3780 | 0.048 | 0.5669 | -0.047 | 0.8926 | 0.013 | 0.4573 | 0.032 | 0.4450 |
| **Pr1_14** | 0.134 | 0.0890 | -0.001 | 0.1954 | 0.033 | 0.1010 | -0.010 | 0.5971 | -0.009 | 0.7431 | -0.002 | 0.7263 | 0.027 | 0.4573 | -0.049 | 0.6342 |
| **Pr1_15** | -0.001 | 0.4207 | 0.019 | 0.0554 | 0.007 | 0.1567 | -0.004 | 0.5599 | -0.033 | 0.7355 | 0.019 | 0.2471 | -0.041 | 0.5783 | 0.002 | 0.5271 |
| **Pr1_16** | -0.027 | 0.6022 | 0.043 | 0.0309 | 0.010 | 0.1512 | 0.079 | 0.3749 | 0.018 | 0.6278 | -0.034 | 0.8926 | -0.043 | 0.7483 | -0.084 | 0.7752 |
| **Pr1_17** | -0.008 | 0.5386 | -0.009 | 0.2259 | -0.003 | 0.2523 | -0.035 | 0.5971 | 0.003 | 0.7034 | 0.007 | 0.5255 | 0.006 | 0.4706 | -0.023 | 0.5271 |
| **Pr1_18** | 0.046 | 0.1735 | **0.040** | 0.0000 | **0.078** | 0.0000 | -0.022 | 0.6175 | 0.034 | 0.5669 | 0.043 | 0.5255 | 0.021 | 0.4573 | 0.034 | 0.3797 |
| **Pr1_19** | -0.004 | 0.4604 | **0.058** | 0.0000 | **0.220** | 0.0000 | 0.070 | 0.2122 | 0.014 | 0.5669 | -0.001 | 0.7765 | 0.091 | 0.3500 | -0.005 | 0.5622 |
| **Pr1_21** | -0.007 | 0.6022 | 0.016 | 0.1041 | **0.059** | 0.0000 | 0.017 | 0.4545 | 0.017 | 0.6700 | -0.025 | 0.8926 | **0.161** | 0.0000 | **0.163** | 0.0653 |
| **Pr1_22** | -0.016 | 0.4821 | **0.058** | 0.0000 | **0.087** | 0.0000 | **0.083** | 0.0422 | 0.077 | 0.4873 | -0.042 | 0.8090 | -0.021 | 0.5783 | 0.055 | 0.3005 |
| **Pr1_24** | -0.026 | 0.6230 | 0.013 | 0.1480 | 0.019 | 0.0833 | 0.101 | 0.2533 | 0.000 | 0.7034 | 0.154 | 0.5255 | 0.066 | 0.3854 | -0.004 | 0.7752 |
| **Pr1_26** | 0.034 | 0.3270 | 0.061 | 0.0309 | 0.127 | 0.0412 | 0.076 | 0.2111 | -0.001 | 0.6212 | -0.001 | 0.7593 | -0.058 | 0.6624 | -0.008 | 0.5271 |
| **Pr1_28** | -0.021 | 0.6230 | 0.012 | 0.1413 | 0.032 | 0.0494 | 0.199 | 0.0704 | -0.013 | 0.7034 | 0.157 | 0.5255 | -0.026 | 0.4485 | **0.070** | 0.2406 |
| **Pr1_29** | 0.016 | 0.3642 | -0.014 | 0.3511 | 0.018 | 0.1525 | 0.014 | 0.5039 | 0.021 | 0.5669 | 0.022 | 0.5862 | **0.101** | 0.2100 | -0.012 | 0.8439 |
| **Pr1_31** | 0.015 | 0.3664 | **0.095** | 0.0129 | 0.127 | 0.0412 | -0.035 | 0.5971 | -0.007 | 0.7034 | -0.031 | 0.5912 | -0.066 | 0.6982 | **0.182** | 0.0941 |
| **Pr1_36** | -0.017 | 0.6230 | 0.006 | 0.1480 | 0.016 | 0.1525 | -0.012 | 0.6175 | -0.010 | 0.7993 | 0.001 | 0.7312 | -0.018 | 0.5783 | 0.110 | 0.0941 |
| **Pr1_40** | **0.174** | 0.0000 | **0.371** | 0.0000 | **0.139** | 0.0000 | 0.063 | 0.2111 | -0.030 | 0.8571 | 0.048 | 0.5255 | 0.197 | 0.0757 | 0.007 | 0.5041 |
| **Pr1_43** | -0.021 | 0.5874 | 0.012 | 0.0922 | 0.032 | 0.0412 | 0.047 | 0.2122 | **0.142** | 0.2419 | 0.022 | 0.5912 | -0.021 | 0.4485 | 0.070 | 0.2933 |
| **Pr1_45** | -0.001 | 0.4650 | **0.074** | 0.0000 | **0.097** | 0.0185 | 0.021 | 0.3780 | -0.041 | 0.9397 | 0.008 | 0.5276 | -0.102 | 0.7465 | 0.014 | 0.4981 |
| **Pr1_46** | **0.247** | 0.0000 | **0.550** | 0.0000 | **0.042** | 0.0185 | **0.117** | 0.0422 | 0.108 | 0.2419 | -0.011 | 0.8090 | -0.048 | 0.7998 | 0.049 | 0.3797 |
| **Pr1_47** | -0.005 | 0.4604 | 0.016 | 0.0309 | -0.008 | 0.2234 | **0.198** | 0.0422 | 0.142 | 0.4873 | -0.043 | 0.8926 | 0.054 | 0.3906 | 0.001 | 0.5015 |
| **Pr1_48** | -0.012 | 0.7835 | 0.014 | 0.1041 | 0.056 | 0.0561 | 0.016 | 0.5039 | 0.020 | 0.5669 | 0.014 | 0.5912 | **0.162** | 0.2100 | -0.005 | 0.5622 |
| **Pr2_3** | -0.015 | 0.5592 | 0.007 | 0.0858 | **0.066** | 0.0185 | -0.011 | 0.5971 | -0.014 | 0.8571 | -0.009 | 0.8050 | 0.060 | 0.3500 | -0.005 | 0.5271 |
| **Pr2_4** | 0.060 | 0.4207 | **0.346** | 0.0000 | **0.205** | 0.0000 | -0.011 | 0.8009 | 0.000 | 0.9414 | 0.000 | 0.8926 | 0.123 | 0.3761 | 0.093 | 0.3797 |
| **Pr2_5** | -0.010 | 0.6022 | 0.009 | 0.1581 | -0.002 | 0.2593 | -0.011 | 0.4776 | 0.017 | 0.6436 | -0.004 | 0.8050 | **0.196** | 0.2048 | **0.117** | 0.0941 |
| **Pr2_7** | -0.005 | 0.4032 | -0.006 | 0.3510 | 0.033 | 0.0760 | 0.017 | 0.4921 | 0.018 | 0.5669 | 0.000 | 0.8926 | -0.042 | 0.6318 | -0.013 | 0.8439 |
| **Pr2_9** | 0.059 | 0.3374 | 0.030 | 0.0309 | 0.032 | 0.1261 | -0.010 | 0.5971 | -0.029 | 0.8571 | -0.017 | 0.8926 | -0.043 | 0.7744 | **0.111** | 0.1108 |
| **Pr2_11** | 0.008 | 0.4207 | -0.013 | 0.3367 | 0.001 | 0.1466 | 0.052 | 0.2533 | 0.052 | 0.4873 | 0.040 | 0.5255 | 0.053 | 0.3662 | -0.027 | 0.7490 |
| **Pr2_12** | -0.018 | 0.5874 | 0.018 | 0.1480 | **0.037** | 0.0000 | -0.014 | 0.6175 | -0.014 | 0.8571 | -0.048 | 0.8926 | -0.036 | 0.6049 | **0.130** | 0.1125 |
| **Pr2_16** | 0.012 | 0.4207 | 0.014 | 0.1324 | 0.008 | 0.1653 | 0.082 | 0.2111 | -0.001 | 0.7034 | 0.009 | 0.6686 | **0.216** | 0.2100 | 0.014 | 0.5006 |
| **Pr2_17** | -0.018 | 0.5386 | 0.001 | 0.2009 | -0.016 | 0.2765 | 0.017 | 0.3780 | 0.048 | 0.5669 | 0.003 | 0.5365 | -0.129 | 0.7915 | 0.010 | 0.5015 |
| **Pr2_20** | -0.005 | 0.5386 | 0.015 | 0.0858 | 0.089 | 0.0309 | -0.016 | 0.6175 | 0.016 | 0.6212 | -0.001 | 0.8218 | -0.104 | 0.6752 | 0.116 | 0.0941 |
| **Pr2_23** | -0.008 | 0.4207 | 0.024 | 0.0858 | -0.006 | 0.1884 | 0.137 | 0.0779 | 0.017 | 0.5669 | 0.004 | 0.7588 | 0.107 | 0.2100 | -0.024 | 0.6207 |
| **Pr2_25** | 0.016 | 0.3664 | 0.030 | 0.0240 | 0.042 | 0.0682 | **0.201** | 0.0422 | 0.029 | 0.5669 | 0.077 | 0.5255 | **0.173** | 0.2100 | -0.031 | 0.6992 |
| **Pr2_28** | -0.002 | 0.4207 | 0.022 | 0.0554 | **0.089** | 0.0185 | -0.040 | 0.5971 | 0.017 | 0.6212 | 0.001 | 0.5255 | 0.011 | 0.4958 | 0.054 | 0.2406 |
| **Pr2_29** | 0.009 | 0.4628 | -0.002 | 0.2218 | 0.020 | 0.0903 | -0.011 | 0.5971 | -0.040 | 0.9062 | -0.001 | 0.8090 | 0.07 | 0.3662 | -0.003 | 0.6226 |
| **Pr2_30** | -0.005 | 0.4032 | -0.012 | 0.2962 | -0.018 | 0.2222 | -0.001 | 0.4921 | -0.037 | 0.8571 | -0.033 | 0.7929 | -0.022 | 0.4573 | -0.008 | 0.7006 |
| **Pr2_32** | -0.010 | 0.6022 | 0.009 | 0.1175 | -0.004 | 0.2315 | 0.000 | 0.5558 | -0.040 | 0.8571 | 0.004 | 0.5255 | -0.009 | 0.5438 | -0.009 | 0.6207 |
| **Pr2_34** | -0.004 | 0.4242 | -0.010 | 0.2332 | -0.027 | 0.3704 | -0.040 | 0.5971 | -0.013 | 0.7431 | 0.000 | 0.8926 | -0.013 | 0.4773 | -0.052 | 0.7006 |
| **Pr2_35** | 0.032 | 0.3270 | **0.074** | 0.0000 | 0.002 | 0.2413 | -0.022 | 0.6175 | -0.009 | 0.7034 | -0.027 | 0.8859 | -0.023 | 0.5735 | 0.070 | 0.1792 |
| **Pr2_38** | 0.006 | 0.3560 | -0.011 | 0.3363 | -0.003 | 0.2234 | 0.022 | 0.3780 | -0.035 | 0.7431 | -0.004 | 0.8926 | 0.082 | 0.3662 | -0.001 | 0.5271 |
| **Pr2_41** | 0.034 | 0.1446 | **0.064** | 0.0000 | 0.066 | 0.0561 | 0.044 | 0.3780 | 0.044 | 0.6700 | -0.052 | 0.8859 | 0.107 | 0.2260 | 0.034 | 0.4450 |
| **Pr2_42** | **0.334** | 0.0000 | **0.396** | 0.0000 | -0.015 | 0.3103 | 0.051 | 0.3690 | -0.018 | 0.7431 | 0.038 | 0.5912 | -0.014 | 0.5321 | 0.035 | 0.4297 |
| **Pr2_44** | **0.157** | 0.0000 | **0.204** | 0.0000 | -0.011 | 0.2523 | 0.071 | 0.0760 | -0.021 | 0.8437 | 0.079 | 0.2471 | **0.591** | 0.0472 | 0.041 | 0.4579 |
| **Pr2_45** | 0.050 | 0.1607 | **0.353** | 0.0000 | **0.218** | 0.0000 | -0.004 | 0.4574 | -0.042 | 0.9062 | -0.004 | 0.5912 | 0.255 | 0.1440 | 0.047 | 0.3856 |
| **Pr2_47** | -0.017 | 0.4693 | -0.001 | 0.1864 | 0.024 | 0.0561 | -0.036 | 0.5971 | -0.012 | 0.7431 | **0.014** | 0.1412 | 0.066 | 0.3662 | -0.002 | 0.6226 |
| **Pr2_48** | -0.019 | 0.6022 | -0.005 | 0.2368 | -0.022 | 0.3103 | -0.037 | 0.6122 | 0.019 | 0.5669 | 0.001 | 0.5912 | 0.112 | 0.3060 | -0.009 | 0.4297 |
| **Pr4_1** | -0.013 | 0.5589 | **0.112** | 0.0129 | **0.080** | 0.0000 | -0.036 | 0.6175 | -0.040 | 0.9062 | -0.047 | 0.8869 | **0.181** | 0.2100 | -0.005 | 0.6226 |
| **Pr4_4** | -0.010 | 0.6022 | **0.032** | 0.0129 | 0.008 | 0.1261 | 0.006 | 0.5269 | -0.024 | 0.8437 | -0.011 | 0.8926 | -0.016 | 0.4573 | 0.012 | 0.3005 |
| **Pr4_5** | -0.009 | 0.4032 | **0.023** | 0.0000 | 0.000 | 0.2222 | 0.116 | 0.0704 | 0.022 | 0.5669 | 0.003 | 0.5255 | 0.092 | 0.3500 | **0.172** | 0.0912 |
| **Pr4_10** | **0.081** | 0.0000 | **0.253** | 0.0000 | 0.031 | 0.0682 | 0.075 | 0.0779 | 0.054 | 0.4873 | -0.030 | 0.8926 | -0.038 | 0.6049 | 0.302 | 0.0653 |
| **Pr4_11** | **0.204** | 0.0000 | **0.482** | 0.0000 | **0.153** | 0.0000 | 0.048 | 0.3694 | 0.103 | 0.5087 | -0.039 | 0.8926 | -0.004 | 0.4573 | -0.019 | 0.6226 |
| **Pr4_12** | -0.013 | 0.5386 | **0.062** | 0.0129 | 0.095 | 0.0654 | 0.017 | 0.4574 | -0.006 | 0.7034 | -0.033 | 0.8599 | 0.040 | 0.4161 | 0.119 | 0.2406 |
| **Pr4_17** | 0.039 | 0.1735 | **0.089** | 0.0000 | 0.095 | 0.0309 | 0.007 | 0.5039 | -0.023 | 0.8437 | 0.001 | 0.7154 | 0.004 | 0.5005 | -0.013 | 0.6459 |
| **Pr4_18** | **0.067** | 0.0000 | 0.002 | 0.3363 | **0.038** | 0.0000 | -0.013 | 0.8009 | -0.013 | 0.9414 | 0.000 | 0.8926 | -0.040 | 0.6049 | 0.000 | 0.8439 |
| **Pr4_19** | **0.057** | 0.0526 | **0.227** | 0.0000 | 0.053 | 0.0682 | -0.020 | 0.6099 | -0.015 | 0.7993 | -0.030 | 0.8859 | 0.098 | 0.3060 | 0.062 | 0.3293 |
| **Pr4_21** | -0.021 | 0.6022 | 0.010 | 0.1480 | 0.027 | 0.1653 | 0.016 | 0.5039 | 0.016 | 0.7034 | 0.008 | 0.6686 | -0.037 | 0.6049 | 0.018 | 0.4721 |
| **Pr4_27** | 0.076 | 0.0890 | 0.016 | 0.1413 | **0.153** | 0.0000 | -0.040 | 0.6175 | -0.011 | 0.7034 | 0.000 | 0.6686 | -0.002 | 0.4573 | **0.131** | 0.0912 |
| **Pr4_34** | -0.022 | 0.6022 | 0.004 | 0.2009 | **0.034** | 0.0000 | -0.012 | 0.5971 | -0.012 | 0.7034 | 0.001 | 0.8859 | **0.124** | 0.0472 | 0.055 | 0.4202 |
| **Pr4_38** | **0.133** | 0.0000 | -0.006 | 0.3558 | 0.093 | 0.0494 | -0.015 | 0.6175 | -0.010 | 0.8253 | 0.016 | 0.5862 | -0.024 | 0.6049 | 0.059 | 0.3454 |
| **Pr4_41** | 0.001 | 0.1607 | **0.033** | 0.0000 | 0.031 | 0.0309 | 0.093 | 0.0779 | 0.018 | 0.5669 | **0.019** | 0.2118 | 0.117 | 0.2100 | 0.006 | 0.5041 |
| **rps4_4** | 0.087 | 0.1157 | **0.088** | 0.0129 | 0.002 | 0.2234 | 0.004 | 0.4088 | -0.020 | 0.7034 | 0.068 | 0.5912 | 0.054 | 0.4154 | 0.001 | 0.5271 |
| **rps10** | -0.009 | 0.4207 | **0.023** | 0.0129 | -0.002 | 0.2222 | -0.013 | 0.6046 | -0.014 | 0.8571 | 0.023 | 0.6652 | 0.063 | 0.4042 | 0.043 | 0.4176 |
| **umn927** | -0.006 | 0.4207 | 0.002 | 0.2259 | 0.028 | 0.1010 | -0.013 | 0.6175 | -0.009 | 0.7034 | -0.033 | 0.8926 | 0.118 | 0.2260 | 0.165 | 0.0869 |
| **175** | 0.055 | 0.4132 | **0.035** | 0.0000 | -0.031 | 0.3216 | -0.003 | 0.5269 | 0.005 | 0.6700 | -0.020 | 0.8926 | 0.121 | 0.2719 | -0.024 | 0.4526 |
| **a3ip** | -0.026 | 0.5874 | -0.006 | 0.2903 | -0.009 | 0.2523 | -0.033 | 0.6621 | 0.082 | 0.4873 | 0.151 | 0.5255 | 0.158 | 0.3500 | -0.063 | 0.7752 |
| **abaR** | -0.024 | 0.6093 | -0.004 | 0.2218 | -0.026 | 0.3210 | **0.083** | 0.0422 | **0.196** | 0.0000 | -0.013 | 0.8379 | **0.581** | 0.0472 | -0.044 | 0.6339 |
| **ccoam** | **0.109** | 0.0526 | 0.025 | 0.1303 | **0.221** | 0.0000 | -0.034 | 0.5971 | 0.082 | 0.4873 | 0.013 | 0.5912 | 0.048 | 0.4485 | -0.035 | 0.6498 |
| **chcs** | 0.042 | 0.3471 | -0.006 | 0.2218 | 0.025 | 0.1070 | -0.028 | 0.5971 | -0.037 | 0.8437 | -0.041 | 0.8050 | -0.077 | 0.5873 | -0.008 | 0.5271 |
| **dhn2pp** | 0.015 | 0.4132 | -0.002 | 0.2151 | 0.063 | 0.0970 | **0.253** | 0.0704 | -0.034 | 0.8437 | 0.171 | 0.5255 | 0.059 | 0.4573 | 0.019 | 0.4721 |
| **eph** | -0.012 | 0.5874 | 0.029 | 0.0309 | 0.003 | 0.1653 | -0.008 | 0.5971 | -0.037 | 0.9397 | -0.040 | 0.8869 | 0.105 | 0.3207 | 0.086 | 0.4061 |
| **phy** | **0.070** | 0.0000 | **0.031** | 0.0000 | -0.020 | 0.3463 | **0.213** | 0.0000 | 0.078 | 0.2419 | 0.033 | 0.5862 | 0.116 | 0.3314 | 0.001 | 0.5041 |
| **phytP** | 0.064 | 0.1157 | **0.102** | 0.0000 | -0.023 | 0.4548 | 0.022 | 0.3780 | -0.039 | 0.8571 | 0.003 | 0.5255 | 0.038 | 0.4458 | 0.028 | 0.4297 |
| **pi0** | **0.8127** |  | **0.506** |  | **0.520** |  | **0.801** |  | **0.942** |  | **0.893** |  | **0.815** |  | **0.843** |  |

**Table S11.** Proportion of true null hypotheses (pi0) for Fst values in pairwise comparisons between taxa at all polymorphic sites. Calculations based on QVALUE method (Storey and Tibshirani 2003).

|  | ***Pinus mugo*** | ***P. uliginosa*** |
| --- | --- | --- |
| ***P. mugo*** |  |  |
| ***P. uliginosa*** | 0.896 |  |
| ***P. uncinata*** | 0.778 | 0.889 |

**Table S12.** Outlier SNPs and indels of highest frequency difference in pairwise comparisons between taxa.

| ***Pinus mugo* (PM) *vs P. uliginosa* (PUG)** | | | | | |  |  |  |  |  |  |
| --- | --- | --- | --- | --- | --- | --- | --- | --- | --- | --- | --- |
| SNP position | | | **PM** | **PUG** |  | SNP position | | | **PM** | **PUG** |  |
| **Pr1_10** | C /G | 203 | C: 0.92 | G: 0.29 | SL | **Pr1_46** | T /C | 482 | T : 0.91 | C : 0.83 | SL |
| **Pr1_21** | G /A | 210 | G: 0.95 | A: 0.42 | SL | **Pr2_42** | A /G | 481 | A: 0.82 | G: 0.67 | SL |
| **Pr1_26** | T /C | 237 | T : 0.90 | C : 0.42 | NS | **Pr2_42** | G /T | 390 | G: 0.82 | T: 0.67 | SL |
| **Pr1_46** | G /A | 171 | G: 0.72 | A: 0.83 | SL | **Pr2_42** | A /G | 382 | A: 0.82 | G: 0.67 | SL |
| **Pr1_46** | A /C | 232 | A: 0.96 | C : 0.75 | SL | **Pr2_42** | T /C | 223 | T: 0.82 | C: 0.67 | SL |
| **Pr1_46** | C /T | 384 | C: 0.96 | T : 0.79 | SL | **Pr4_10** | G /A | 129 | G: 0.92 | A: 0.50 | SL |
| **Pr1_46** | T /C | 395 | T : 0.98 | C : 0.67 | SL | **phy** | G /A | 185 | G: 0.92 | A: 0.50 | SL |
| **Pr1_46** | A /C | 447 | A: 0.91 | C : 0.83 | SL |  |  |  |  |  |  |
| ***P. mugo*  (PM) *vs. P. uncinata* (PUN)** | | | | | |  |  |  |  |  |  |
| SNP position | | | **PM** | **PUN** |  | SNP position | | | **PM** | **PUN** |  |
| **Pr1_10** | C /A | 271 | C : 0.94 | A: 0.46 | SL | **Pr2_23** | A /G | 356 | A: 0.75 | G: 0.82 | SL |
| **Pr1_22** | T /C | 293 | T : 0.68 | C : 0.86 | NS | **Pr2_42** | G/T | 390 | G: 0.82 | T: 0.74 | SL |
| **Pr1_22** | C /G | 224 | C : 0.94 | G: 0.38 | NS | **Pr2_42** | A /G | 481 | A: 0.82 | G: 0.74 | SL |
| **Pr1_28** | G /T | 70 | G: 0.81 | T : 0.68 | NS | **Pr2_42** | A/G | 382 | A: 0.82 | G: 0.74 | SL |
| **Pr1_28** | A /G | 247 | A: 0.77 | G: 0.96 | SL | **Pr2_42** | T/C | 223 | T: 0.82 | C: 0.74 | SL |
| **Pr1_40** | A /C | 13 | A: 0.63 | C : 0.94 | SL | **Pr2_44** | A /T | 110 | A: 0.92 | T : 0.42 | SL |
| **Pr1_46** | A /C | 232 | A: 0.96 | C : 0.70 | SL | **Pr2_45** | T/- | 189 | T:0.40 | -:1.00 |  |
| **Pr1_46** | C /T | 384 | C : 0.96 | T : 0.70 | SL | **Pr2_45** | C /T | 261 | C: 0.77 | T: 0.80 | SL |
| **Pr1_46** | A /C | 447 | A: 0.91 | C : 0.90 | SL | **Pr2_45** | A/T | 188 | A: 0.77 | T: 0.80 | SL |
| **Pr1_46** | T /C | 482 | T : 0.91 | C : 0.88 | SL | **Pr4_11** | T /C | 60 | T : 0.75 | C : 0.78 | SL |
| **Pr2_23** | T /G | 81 | T : 0.79 | G: 0.84 | NS | **Pr4_11** | G /A | 361 | G: 0.65 | A: 0.90 | SL |
| **Pr2_23** | G /A | 326 | G: 0.80 | A: 0.74 | SL |  |  |  |  |  |  |

| ***P. uliginosa* (PUG) *vs P. uncinata* (PUN)** | | | | | |  |  |  |  |  |  |
| --- | --- | --- | --- | --- | --- | --- | --- | --- | --- | --- | --- |
| **SNP position** | | | **PUG** | **PUN** |  | **SNP position** | | | **PUG** | **PUN** |  |
| Pr1_10 | C /A | 271 | C:0.92 | A:0.46 | SL | Pr4_4 | G /A | 451 | G:0.58 | A:0.82 | SL |
| Pr1_21 | A /G | 210 | A:0.42 | G:0.96 | SL | Pr4_4 | A /C | 164 | A:0.58 | C:0.82 | SL |
| Pr1_28 | G /T | 70 | G:0.88 | T:0.68 | NS | Pr4_4 | A /T | 163 | A:0.58 | T:0.82 | SL |
| Pr1_28 | A /G | 247 | A:0.67 | G:0.96 | SL | chcs | C /T | 12 | C:1.00 | T:0.64 | SL |
| Pr2_45 | T /- | 189 | T:0.33 | -:1.00 | SL | chcs | A /G | 42 | A:1.00 | G :0.56 | SL |

**Table S13.** Outlier SNPs detected across loci in pairwise comparisons between geographical groups determined for *Pinus mugo* and three and five populations of *P. uliginosa* and *P. uncinata*, respectively.

| ***Pinus mugo*: 3 proups** | | | | | |  |  |  |  |  |  |
| --- | --- | --- | --- | --- | --- | --- | --- | --- | --- | --- | --- |
|  | **SNPs position** | | **Fst** | **p** |  |  | **SNPs position** | | **Fst** | **p** |  |
| Pr1_9 | 232 | T/A | 0.229 | 0.016 | NS | Pr2_16 | 223 | T/C | 0.232 | 0.010 | SL |
| Pr1_15 | 504 | A/G | 0.228 | 0.011 | SL | Pr2_25 | 119 | G/A | 0.212 | 0.017 | SL |
| Pr1_15 | 497 | A/C | 0.228 | 0.011 | SL | Pr2_41 | 83 | A/G | 0.138 | 0.050 | SL |
| Pr1_15 | 332 | A/G | 0.228 | 0.011 | SL | Pr2_48 | 232 | C/A | 0.129 | 0.041 | SL |
| Pr1_15 | 219 | G/A | 0.177 | 0.028 | SL | Pr4_5 | 31 | T/C | 0.176 | 0.026 | SL |
| Pr1_15 | 229 | G/T | 0.131 | 0.037 | SL | Pr4_12 | 512 | A/C | 0.193 | 0.019 | SL |
| Pr1_15 | 211 | T/A | 0.228 | 0.011 | SL | Pr4_41 | 229 | G/A | 0.121 | 0.048 | NS |
| Pr1_17 | 294 | C/A | 0.153 | 0.036 | SL | Pr4_41 | 457 | C/G | 0.267 | 0.006 | SL |
| Pr1_19 | 110 | A/G | 0.121 | 0.048 | NA | abaR | 37 | G/A | 0.147 | 0.039 | SL |
| Pr1_22 | 187 | A/G | 0.249 | 0.009 | NS | chcs | 75 | A/C | 0.162 | 0.044 | SL |
| Pr1_22 | 71 | T/C | 0.238 | 0.010 | SL | chcs | 111 | G/C | 0.212 | 0.030 | SL |
| Pr1_31 | 194 | G/A | 0.186 | 0.019 | NS | dhn2pp | 278 | T/C | 0.171 | 0.040 | SL |
| Pr1_46 | 171 | G/A | 0.222 | 0.015 | SL | phy | 158 | A/G | 0.178 | 0.039 | SL |
| Pr2_11 | 242 | C/T | 0.237 | 0.009 | SL |  |  |  |  |  |  |
| ***P. uliginosa*: 3 populations** | | | | | |  |  |  |  |  |  |
| Pr1_12 | 171 | G/A | 0.515 | 0.029 | NS | Pr4_1 | 17 | T/C | 0.269 | 0.023 | NA |
| Pr1_12 | 38 | T/G | 0.274 | 0.000 | SL | Pr4_1 | 288 | C/G | 0.428 | 0.018 | NA |
| Pr1_29 | 248 | A/T | 0.106 | 0.020 | SL | Pr4_1 | 328 | G/A | 0.428 | 0.018 | NA |
| Pr1_40 | 82 | G/A | 0.382 | 0.036 | SL | Pr4_4 | 283 | A/G | 0.471 | 0.017 | SL |
| Pr1_47 | 396 | G/C | 0.467 | 0.035 | SL | Pr4_4 | 239 | G/A | 0.471 | 0.017 | SL |
| Pr2_44 | 224 | T/C | 0.503 | 0.039 | NA | Pr4_4 | 134 | A/T | 0.471 | 0.017 | SL |
| ***P. uncinata*: 5 populations** | | | | | |  |  |  |  |  |  |
| Pr1_11 | 100 | T/C | 0.284 | 0.020 | SL | umn927 | 335 | G/T | 0.283 | 0.013 | SL |
| Pr4_5 | 315 | T/A | 0.235 | 0.039 | SL | umn927 | 336 | A/T | 0.283 | 0.013 | SL |
| Pr4_5 | 290 | G/A | 0.235 | 0.039 | SL | abaR | 350 | C/T | 0.163 | 0.034 | SL |
| Pr4_5 | 169 | T/C | 0.235 | 0.039 | SL | dhn2pp | 293 | G/T | 0.235 | 0.038 | SL |
| Pr4_27 | 224 | T/C | 0.250 | 0.032 | SL |  |  |  |  |  |  |

NS – nonsynonymous site, SL-silent site, NA-coding regions not annotated

**Supplementary Figures**

**Fig S1.** Nucleotide diversity (_total_) at 79 loci in the three pine taxa studied (locus number in reference to S2 Table).

*
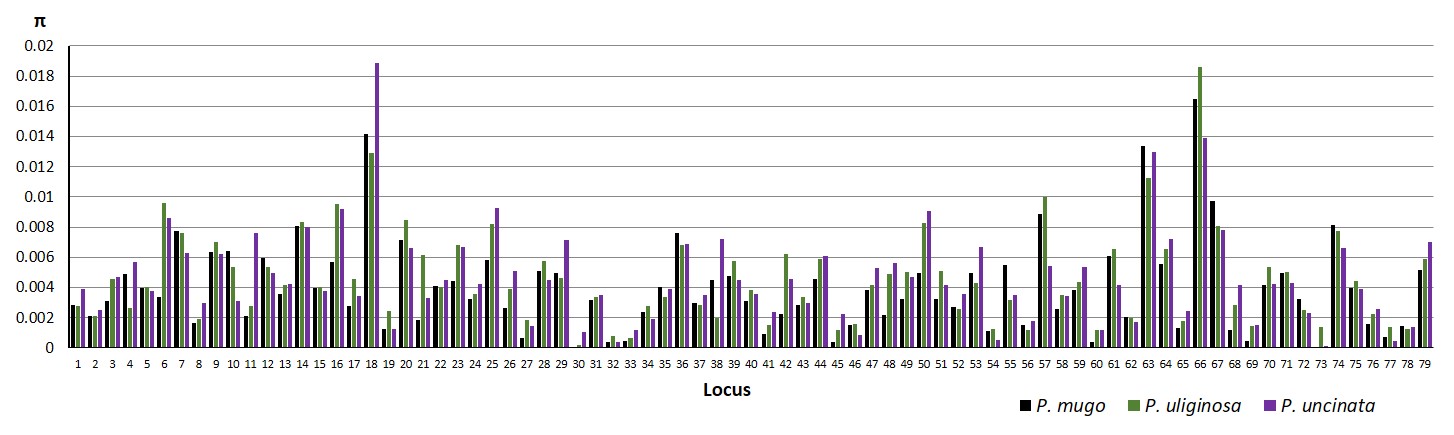
*

**Fig S2.** Net divergence (Da) at 79 analysed loci in pairwise comparisons between *Pinus mugo*, *P. uliginosa* and *P. uncinata*. Gene acronyms as in Table 3 and Supplementary Table S2.


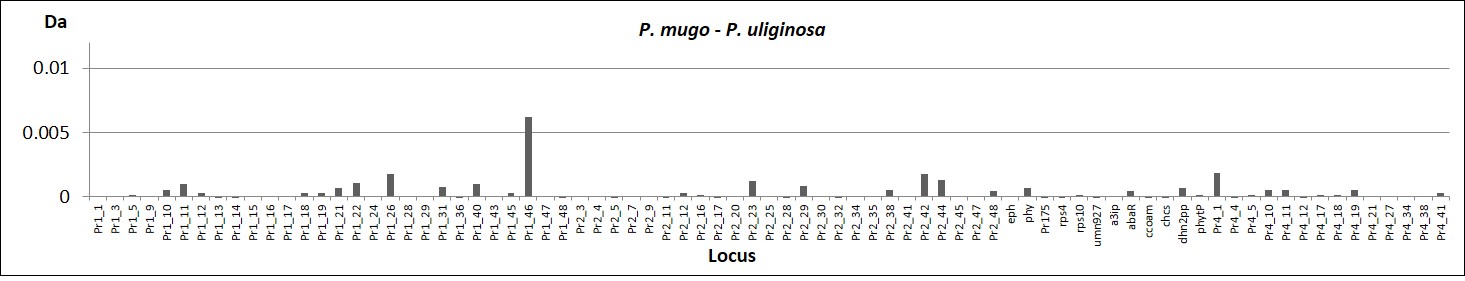


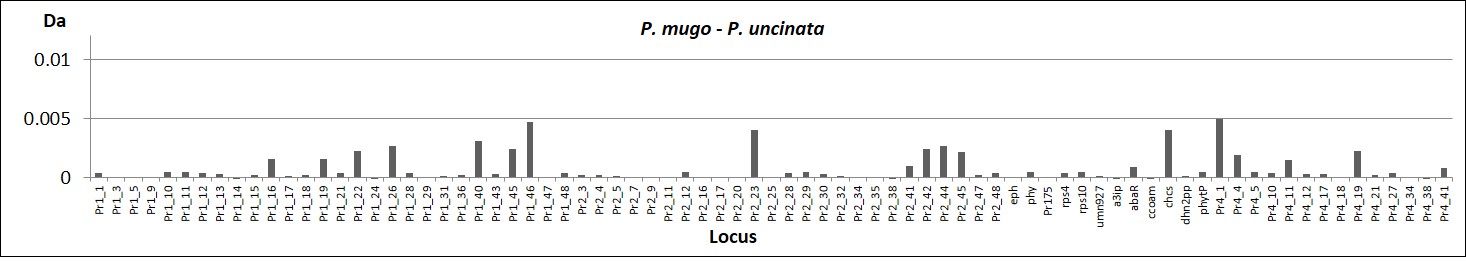

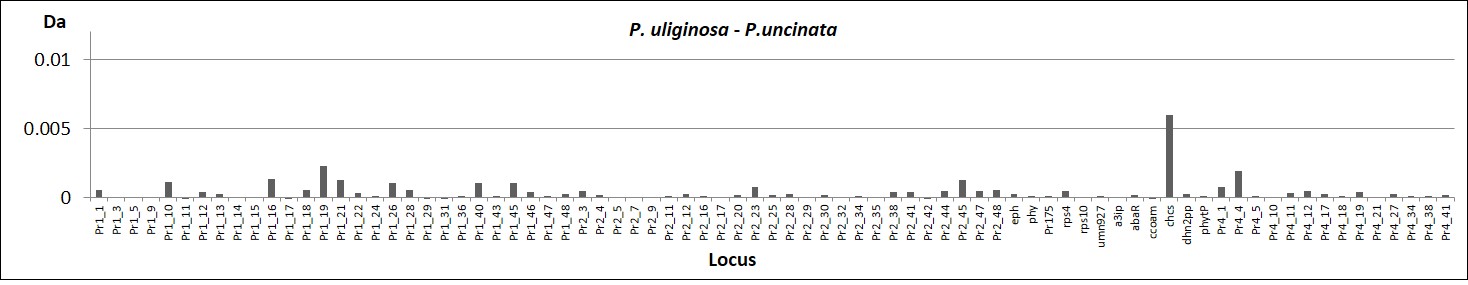


**Fig S3.** Scatter plot of the squared correlation coefficient of allele frequencies (r^2^) as a function of distance in base pairs between pairs of polymorphic sites in *Pinus mugo* (A), *P. uliginosa* (B) and *P. uncinata* (C) at all 79 nuclear loci combined. Decline in linkage disequilibrium is shown by nonlinear fitting curve of the mutation-recombination-drift model (see material and methods section for details). Recombination rate parameter ρ (standard error in parenthesis) for *P. mugo* is ρ=0.0345 (0,0026), for *P. uliginosa* is ρ = 0.0028 (0.0005) and for *P. uncinata* is ρ =0.0184 (0.0015).

**A. B. C.**

**APPENDIX 1.**

(A) Results of the Bayesian clustering analysis with BAPS software based on a sequence variation at 79 loci for the three taxa from the *Pinus mugo* complex.

Analysis KL-divergence matrix in PHYLIP format:

Cluster_1 0.000 0.152

Cluster_2 0.152 0.000

List of sizes of 10 best visited partitions and corresponding log (ml) values

2 -26854.15

2 -26854.4535

2 -26855.2619

2 -26855.5481

2 -26855.6598

2 -26855.8952

2 -26855.8974

2 -26855.9061

2 -26856.9117

2 -26856.9477

Probabilities for number of clusters

2 1

(B) Results based on clustering analysis with STRUCTURE (Pritchard et al. 2000) for the three taxa from the *Pinus mugo* complex. LnP(K) values were relatively stable across K=1-6 so delta K was misleading as a means of identifying the optimal value of K. Inspection of cluster assignments at different K values clearly indicated that K=2 was the optimal value, with additional clusters always minor (see plot for K=3, below). PM – *P. mugo*, PUG – *P. uliginosa*, PUN – *P. uncinata*.

| **K** | **Reps** | **Mean LnP(K)** | **Stdev LnP(K)** | **Ln'(K)** | **\|Ln''(K)\|** | **Delta K** |
| --- | --- | --- | --- | --- | --- | --- |
| 1 | 3 | -40079.766667 | 82.501657 | — | — | — |
| 2 | 3 | -37917.333333 | 1410.669041 | 2162.433333 | 1519.000000 | 1.076794 |
| 3 | 3 | -37273.900000 | 58.170181 | 643.433333 | 1150.800000 | 19.783332 |
| 4 | 3 | -37781.266667 | 707.464666 | -507.366667 | 954.600000 | 1.349325 |
| 5 | 3 | -39243.233333 | 3384.432463 | -1461.966667 | 2444.900000 | 0.722396 |
| 6 | 3 | -43150.100000 | 1998.485084 | -3906.866667 | 95930.400000 | 48.001559 |
| 7 | 3 | -142987.366667 | 175503.439669 | -99837.266667 | 128485.100000 | 0.732094 |
| 8 | 3 | -371309.733333 | 302948.274517 | -228322.366667 | 208568.833333 | 0.688464 |
| 9 | 3 | -391063.266667 | 282016.091124 | -19753.533333 | 14487.166667 | 0.051370 |
| 10 | 3 | -425303.966667 | 667041.636399 | -34240.700000 | — | — |

K=2


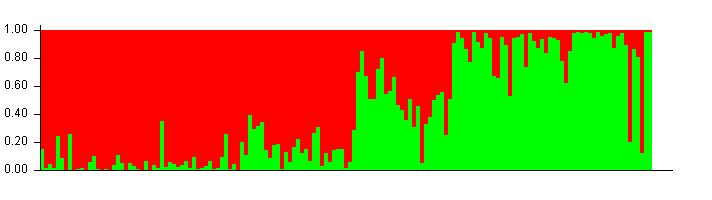

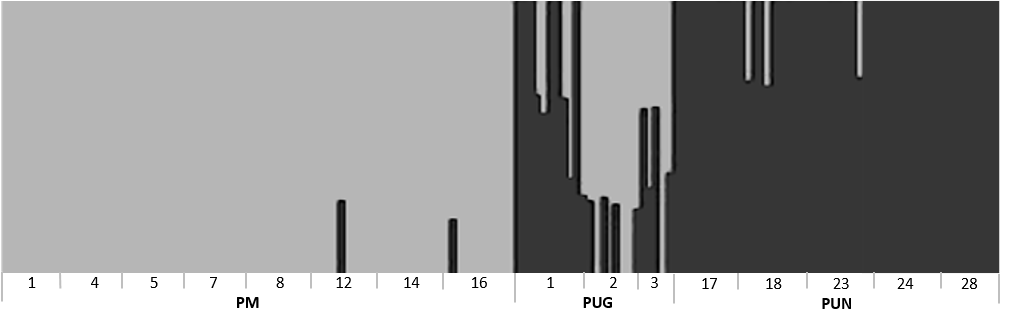


K=3


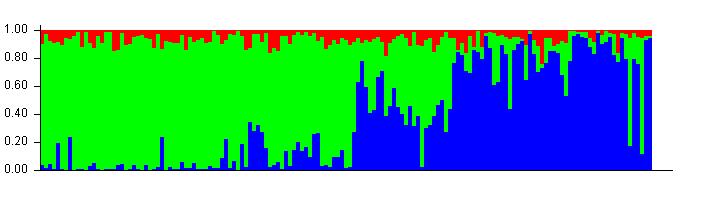

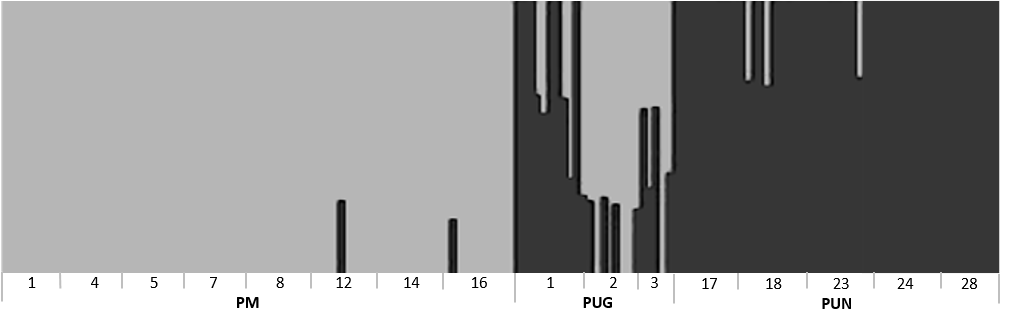


**References**

Corander J, Tang J (2007) Bayesian analysis of population structure based on linked molecular information. Math Biosci 205:19-31

Donnelly K et al. (2017) Reconstructing the plant mitochondrial genome for marker discovery: a case study using Pinus. Mol Ecol Resour 17:943-954

Ersoz ES, Wright MH, González-Martínez SC, Langley CH, D.B. N (2010) Evolution of disease response genes in loblolly pine: insights from candidate genes. PLoS One 5:e14234 doi:10.1371/journal.pone.0014234

Eveno E et al. (2008) Contrasting patterns of selection at *Pinus pinaster* Ait. drought stress candidate genes as revealed by genetic differentiation analyses. Mol Biol Evol 25:417 - 437

Hudson RR, Kreitman M, Aguade M (1987) A test of neutral molecular evolution based on nucleotide data. Genetics 116:153-159

Palmé AE, Wright M, Savolainen O (2008) Patterns of divergence among conifer ESTs and polymorphism in *Pinus sylvestris* identify putative selective sweeps. Mol Biol Evol 25(12): 2567-2577

Pritchard JK, Stephens M, Donnelly P (2000) Inference of population structure using multilocus genotype data. Genetics 155:945-959

Pyhäjärvi T, Garcia-Gil MR, Knürr T, Mikkonen M, Wachowiak W, Savolainen O (2007) Demographic history has influenced nucleotide diversity in European *Pinus sylvestris* populations. Genetics 177:1713-1724

Storey JD, Tibshirani R (2003) Statistical significance for genomewide studies. Proc Nat Acad Sci USA 100:9440-9445

Wachowiak W, Balk P, Savolainen O (2009) Search for nucleotide diversity patterns of local adaptation in dehydrins and other cold-related candidate genes in Scots pine (*Pinus sylvestris* L.). Tree Genet Genomes 5:117-132
